# Adherence to Direct Oral Anticoagulants in Patients With Non-Valvular Atrial Fibrillation: A Cross-National Comparison in Six European Countries (2008–2015)

**DOI:** 10.3389/fphar.2021.682890

**Published:** 2021-11-03

**Authors:** M. Sabaté, X. Vidal, E. Ballarin, M. Rottenkolber, S. Schmiedl, B. Grave, C. Huerta, E. Martin-Merino, D. Montero, L. M. Leon-Muñoz, C. Gasse, N. Moore, C. Droz, R. Lassalle, M. Aakjær, M. Andersen, M. L. De Bruin, P. Souverein, O. H. Klungel, H. Gardarsdottir, L. Ibáñez

**Affiliations:** ^1^ Fundació Institut Català de Farmacologia (FICF), Barcelona, Spain; ^2^ Department of Clinical Pharmacology, Hospital Universitari Vall d'Hebron, Barcelona, Spain; ^3^ Department of Pharmacology, Toxicology and Therapeutics, Universitat Autònoma de Barcelona, Barcelona, Spain; ^4^ Diabetes Research Group, Medizinische Klinik und Poliklinik IV, Klinikum der Universität München, Munich, Germany; ^5^ Department of Clinical Pharmacology, School of Medicine, Faculty of Health, Witten/Herdecke University, Witten, Germany; ^6^ Philipp Klee-Institute for Clinical Pharmacology, Helios University Hospital Wuppertal, Wuppertal, Germany; ^7^ AOK NORDWEST, Dortmund, Germany; ^8^ Pharmacoepidemiology and Pharmacovigilance Division, Spanish Agency of Medicines and Medical Devices (AEMPS), Madrid, Spain; ^9^ Aarhus University, Aarhus, Denmark; ^10^ Bordeaux PharmacoEpi, INSERM CIC1401, Université de Bordeaux, CHU de Bordeaux, Bordeaux, France; ^11^ Pharmacovigilance Research Centre, Department of Drug Design and Pharmacology, Faculty of Health and Medical Sciences, University of Copenhagen, Copenhagen, Denmark; ^12^ Copenhagen Centre for Regulatory Science (CORS), Department of Pharmacy, Faculty of Health and Medical Sciences, University of Copenhagen, Copenhagen, Denmark; ^13^ Division of Pharmacoepidemiology and Clinical Pharmacology, Utrecht Institute for Pharmaceutical Sciences, Faculty of Science, Universiteit Utrecht, David de Wiedgebouw, Utrecht, Netherlands; ^14^ Julius Center, University Medical Center Utrecht, Utrecht, Netherlands; ^15^ Department of Clinical Pharmacy, University Medical Center Utrecht, Utrecht, Netherlands; ^16^ Faculty of Pharmaceutical Sciences, University of Iceland, Reykjavik, Iceland

**Keywords:** adherence, persistence, anticoagulants, non valvular atrial fibrillation, cardiovascular, drug utilization, pharmacoepidemiology, europe

## Abstract

**Aims:** To describe and compare the adherence to different direct oral anticoagulants (DOACs) in eight European databases representing six countries.

**Methods:** Longitudinal drug utilization study of new users (≥18 years) of DOACs (dabigatran, rivaroxaban, apixaban) with a diagnosis of non-valvular atrial fibrillation (2008–2015). Adherence was examined by estimating persistence, switching, and discontinuation rates at 12 months. Primary non-adherence was estimated in BIFAP and SIDIAP databases.

**Results:** The highest persistence rate was seen for apixaban in the CPRD database (81%) and the lowest for dabigatran in the Mondriaan database (22%). The switching rate for all DOACs ranged from 2.4 to 13.1% (Mondriaan and EGB databases, respectively). Dabigatran had the highest switching rate from 5.0 to 20.0% (Mondriaan and EGB databases, respectively). The discontinuation rate for all DOACs ranged from 16.0 to 63.9% (CPRD and Bavarian CD databases, respectively). Dabigatran had the highest rate of discontinuers, except in the Bavarian CD and AOK NORDWEST databases, ranging from 23.2 to 64.6% (CPRD and Mondriaan databases, respectively). Combined primary non-adherence for examined DOACs was 11.1% in BIFAP and 14.0% in SIDIAP. There were differences in population coverage and in the type of drug data source among the databases.

**Conclusion:** Despite the differences in the characteristics of the databases and in demographic and baseline characteristics of the included population that could explain some of the observed discrepancies, we can observe a similar pattern throughout the databases. Apixaban was the DOAC with the highest persistence. Dabigatran had the highest proportion of discontinuers and switchers at 12 months in most databases (EMA/2015/27/PH).

## Introduction

Direct oral anticoagulants (DOACs) have been approved since 2011 to prevent stroke and systemic embolism in patients with non-valvular atrial fibrillation (NVAF). However, they have been commercialized for other indications such as treatment of deep vein thrombosis and pulmonary embolism or prevention of thrombosis after hip/knee replacement since 2008. The first DOAC that entered the EU market was dabigatran (thrombin inhibitor), followed by rivaroxaban, apixaban, and edoxaban (factor Xa inhibitors).

Treatment with DOACs as well as warfarin has been demonstrated to be effective in reducing the stroke incidence and mortality (except for rivaroxaban) in patients with NVAF (Kirchhof et al., 2016; Bai et al., 2017; Proietti et al., 2018). The easier dosing regimens with DOACs, without need for regular monitoring and with fewer interactions, could theoretically improve treatment adherence. However, some concerns have been raised about patients remembering to take DOACs in the absence of blood monitoring (Rodriguez, et al., 2013).

Although the use of DOACs has increased in recent years in Europe (Hanemaaijer et al., 2015; Giner-Soriano et al., 2016), concerns have been raised about the potential impact of no monitoring (no need for International Normalized Ratio [INR] tests) and the influence of multi-morbidity and polypharmacy on DOAC adherence. This lack of adherence could result in a higher incidence of thromboembolic and hemorrhagic complications which would decrease their effectivity and might decrease their safety advantages compared to vitamin K antagonists (VKA) (Rodriguez et al., 2013; Solla-Ruiz et al., 2019). The lack of antidotes in cases of major bleeding was another issue raised when DOACs were first introduced, however, idarucizumab and adexanet alfa were approved in 2015 and 2019, respectively (EMA, 2015; EMA, 2019)*.*

Interest in medication adherence, understood as patients taking their medication as prescribed, has increased in recent years because it may compromise the effectiveness and safety of the drug use. Few studies have measured primary adherence to DOACs, defined as patients failing to collect the first prescription of a medication from the pharmacy, with one study estimating non-adherence to be around 11% (Vrijens et al., 2012; Rodriguez-Bernal et al., 2018).

DOAC treatment persistence has been evaluated in some of the pivotal clinical trial studies. Treatment persistence ranged from 14.5% at 1 year to 25.3% during the whole study period (median follow-up: 1.8 years) (Rodriguez, et al., 2013). In the Re-LY trial, the discontinuation rate at 1 year for dabigatran was 15.5 and 10.2% for warfarin (Connolly et al., 2009). In the ROCKET AF study, the proportion of patients who permanently stopped their assigned therapy before an end-point event (stroke (ischemic or hemorrhagic), systemic embolism, or death from cardiovascular causes) and before the study end was 23.7% in the rivaroxaban group and 22.2% in the warfarin group (Patel et al., 2011; Di Minno et al., 2014). This might represent a limitation for the extrapolation of efficacy study results to clinical practice, as efficacy might be lower in non-adherent patients.

The treatment persistence of DOACs has also been evaluated in real-world clinical practice. Published studies carried out in the United Kingdom, United States, Sweden, and Australia, showed that treatment persistence was higher with DOACs than with VKA (Coleman et al., 2016; Forslund et al., 2016; Martinez et al., 2016; Simons et al., 2016). A cohort study in the Clinical Practice Research Datalink (CPRD) database, showed that persistence at 1 year was significantly higher for DOACs (79.2%) than for VKA (63.6%), and that this alone could lead to fewer cardioembolic strokes (Martinez et al., 2016) as was seen in another Sweden observational study where the DOACs’ persistence was 70% and non-persistence patients had an OR = 2 of stroke and transient ischemic attack (Komen et al., 2020). However, in another study performed on the CPRD, no evidence of a difference in non-persistence between VKA and apixaban [HR 0.92 (95% CI 0.68 ti 1.23)] was found, and non-persistence was higher with dabigatran [HR 1.67 (1.20 to 2.32)] and rivaroxaban [HR 1.41 (1.02 to 1.93)] than apixaban (Johnson et al., 2016).

Other studies performed in claims databases (Coleman et al., 2016; Simons et al., 2016) or administrative registries (Forslund et al., 2016) report on differences in adherence for patients using different DOACs. Users of rivaroxaban had a higher persistence and a lower rate of discontinuation at 2 years, compared with those using dabigatran and warfarin (Coleman et al., 2016; ; Forslund et al., 2016). Differences have been found in the hazard ratios for risk of discontinuation among the different types of DOACs with a higher persistence with warfarin and apixaban than with dabigatran or rivaroxaban [rivaroxaban vs apixaban, 2.14 (1.47–3.11); dabigatran vs apixaban, 1.99 (1.38–2.87); rivaroxaban vs dabigatran, 1.09 (0.89–1.36)] (Simons et al., 2016). There are other studies performed with claims databases [French health insurance system database (SNIIRAM); Foundation for Pharmaceutical Statistics (SFK)]. The French study showed a higher adjusted 1-year discontinuation rate for dabigatran than for VKA new users [36.8 vs 30.2%; 3.0% (1.9–4.1)] (Maura et al., 2017) (Maura et al., 2018). And for one performed with the SFK, non-persistence at 1 year to DOAC was 34% higher compared to VKA (22%) (Zielinski et al., 2019).

The present cross-national study aims to describe the adherence to different DOACs across six European countries using longitudinal data collected in eight electronic health care databases over a period of 8 years.

This adherence drug utilization study is part of a protocol that was developed under the EMA Framework service contract (nr. EMA/2015/27/PH) with regard to the re-opening of competition no. 3.

## Methods

### Setting and Study Population

A longitudinal drug utilization study of new users (≥18 years) of DOACs (dabigatran, rivaroxaban, apixaban; ATC codes: B01AE07, B01AF01, and B01AF02, respectively) with a diagnosis of NVAF (see codes in Supplementary Table S1) was conducted between January 2008 and December 2015 in eight data sources from six European countries (Table 1).

**TABLE 1:** Database characteristics.

	Mondriaan	Danish national registries	AOK NORDWEST^3^	Bavarian claims	BIFAP^a^	SIDIAP	CPRD^a^	EGB^a^
**Source population**	0.4 m	5.5 m	2.7 m	10.5 m	7.5 m	5.8 m	12.5 m	0.7 m
**Year(s) covered for this study**	2012–2015	2008–2015	2008–2015	2008–2015	2008–2015	2009–2015	2008–2015	2013–2015
**Type of database** **^b^**	GP prescribing data	Dispensing data from community pharmacies	Claims database including data for dispensed reimbursed drugs	Claims database including data for dispensed reimbursed drugs	General practice prescribing data/reimbursed data	General practice prescribing data/reimbursed data	General practice prescribing data	Claims database including dispensed reimbursed data
**Data available since**	1991	1994	2007	2008	2001	2006	1987	2004
**Demographic variables available**								
Date of registration	Yes	Yes	Yes	Yes (first consultation)	Yes	Yes	Yes	Yes
Date of transferring out	Yes	Yes	Yes	Yes (last consultation)	Yes	Yes	Yes	Yes
Date of birth	DD-MM-YY	MM-YY	MM-YY	MM-YY	MM-YY	MM-YY	MM-YY	MM-YY
Gender	Yes	Yes	Yes	Yes	Yes	Yes	Yes	Yes
**Drug information available**								
Active international coding	ATC	ATC	ATC	ATC	ATC	ATC	BNF	ATC
Product coding	HPK	Nordic Article Number	PZN	PZN	CNF	Yes^c^	Product code	CIP-13

	Mondriaan	Danish national registries	AOK NORDWEST^3^	Bavarian claims	BIFAP^a^	SIDIAP	CPRD^a^	EGB^a^
Date of prescribing/dispensing	Yes	Yes	Primary care sector: Yes Secondary care sector: Yes (only for a few selected (expensive) compounds, but no (D)OACs prescriptions)	Yes (for each prescription the quarter is documented)	Yes	Yes^d^	Yes	Yes
Quantity prescribed/dispensed	Yes	Yes	Yes (package size)	Yes (package size)	Yes	Yes^e^	Yes	Yes
Dosing regimen	No	No	No	No	Yes	Yes^f^	Yes	No
**Outcome information**								
Outpatient primary care diagnosis	ICPC	No	ICD-10-GM (quarterly base)	ICD-10-GM (for each diagnosis the quarter is documented)	ICPC-2, ICD-9	ICD-10	ICD-9, ICD-10	ICD-10
Hospital discharge diagnosis	No	ICD-8, ICD-10	ICD-10-GM	No	No	No^g^	ICD-9, ICD-10	ICD-10
Laboratory tests	Yes	No	No	No	Yes (as requested by GP)	Yes	Yes	No
Mortality	Yes	Yes	No (incompletely recorded, e.g., no follow-up for patients leaving the AOK)	No	Yes (no cause of death)	Yes (no cause of death)	Yes	Yes (no cause of death)

a Representative of general population.

b Prescribing databases: collected information on prescribed drugs by GP; Reimbursement databases: collected information on dispensed drugs funded by Health Care Services; Dispensing databases: collected all dispensation of prescription drugs regardless of the drug’s reimbursement status Elseviers et al., 2016.

c is registered but not available for research due to confidentiality reasons.

d For dispensing: MM/YY.

e Number of reimbursed packages.

f Only in prescribing data.

g Available for 28% of included population.

A common protocol was applied for data extraction and analysis for each database (EU PASS Register No: 16,014) (EU PAS Register, 2021).

New users were defined as patients ≥18 years initiating a DOAC with a diagnosis of NVAF during the study period, without any use of DOACs for at least 12 months prior to the index date (the day of the first DOAC prescription for each patient during the study period). Patients with previous use of VKA were included. A complete flow chart showing patient inclusion is available in a previous publication (Ibáñez et al., 2019) and in the Supplementary Material.

Patients registered in the database less than 12 months before the index date and patients with a history of valvular atrial fibrillation (see codes in Supplementary Table S1) on or at any time before the index date were excluded.

### Data Sources

Data were retrieved from the following eight databases: 1) the Dutch Mondriaan project, which includes the Julius General Practitioner Network (JHN) database (Smeets et al., 2018); 2) the Danish National Registries (DNR), which includes the Danish National Patient Register, Danish National Prescription Registry, and Danish Civil Registration System (Schmidt et al., 2015; Kildemoes et al., 2011; Schmidt et al., 2014); 3) the AOK NORDWEST database, Germany (Jaunzeme et al., 2013; ^,^ Hoffmann and Koller, 2015); 4) the Bavarian Association of Statutory Health Insurance Physicians database, Germany, referred to here as Bavarian CD, (Mehring et al., 2017); 5) the ‘base de datos para la Investigación Farmacoepidemiológica en Atención Primaria’ (BIFAP), Spain (de Abajo et al., 2013); 6) the Information System for the Development of Research in Primary Care (SIDIAP), Catalonia, Spain (García-Gil et al., 2011); 7) the Clinical Practice Research Datalink (CPRD), United Kingdom (Williams et al., 2012; ^,^ Herrett et al., 2015); and 8) the ‘Echantillon Généraliste de Bénéficiaires’ (EGB), France (Bezin et al., 2017). The characteristics of the databases are described in Table 1 and in a previous publication (Ibáñez et al., 2019).

### Ethical Approval

Investigators in each country had study approval from the corresponding data owners. There were no other requirements since anonymized data were used. Additionally, the main study protocol was revised and approved by an internal EMA panel of experts.

### Outcome

The main outcome was assessing the adherence of DOAC users through different indicators such as persistence rate, switching rate, and discontinuation rate. For the analysis of these indicators we used the treatment episodes defined as a series of subsequent prescriptions or dispensations for a DOAC including a permissible gap of 30 days following the theoretical end date of the DOAC, independent of dose changes and constructed according to the method of Gardarsdottir (Gardarsdottir et al., 2010). In case a subsequent prescription for the same drug was collected before the theoretical end date of a previous prescription, the number overlapping days was added to the theoretical end date of the subsequent prescription.

Adherence was assessed on two levels. We first assessed primary non-adherence which was defined as all patients who received a first DOAC prescription and did not refill the prescribed DOAC in the following 12 months.

For secondary adherence, we assessed a number of usage patterns, including:

- Persistent patients were defined as those still using DOAC at various predefined points in time after treatment initiation (3, 6, 12 months).
- Switchers were defined as patients with a subsequent prescription within the first treatment episode that included another type of DOAC or other oral anticoagulant (at least one prescription of antithrombotic drug (ATC code: B01AA [VKA], B01AE [direct thrombin inhibitors], or B01AF [direct factor Xa inhibitors]).
- Discontinuers were defined as patients that did not receive a subsequent DOAC within 30 days following the theoretical end date of a prior DOAC.

Follow-up of each patient covered the time between the index date until switch of therapy, discontinuation of therapy, or the end of the study, whichever came first.

The previously mentioned definitions were based on the terminology proposed by the European Society for Patient Adherence, Compliance, and Persistence (Vrijens et al., 2012; Helmy et al., 2017).

### Analysis

The analysis was performed using the first treatment episode, and stratified by database and individual DOAC at 12 months after the index date and by sex and age for the whole study period.

Primary non-adherence was only performed in the SIDIAP and BIFAP databases on a sample where both prescriptions and dispensing could be linked for the individual patient. In BIFAP, since 2011 onwards and progressively implemented, currently only 14.4% of all prescriptions could be linked to pharmacy dispensations while all prescriptions in SIDIAP could. These were the only participating databases with available information.

- The persistence rate was estimated as the proportion of patients still on treatment at 3, 6, and 12 months (deceased patients were censored). The log-rank test was applied for analyzing differences in the time until discontinuation between the individual DOACs and specific subgroups of patients (chronic kidney disease [CDK] by diagnosis codes, see Supplementary Table S2). This information was available for all databases, except for the Bavarian Claims database.
- The switching rate of all DOACs at 12 months was calculated as the number of patients switching treatment divided by the total number of patients included in the study population *100. The switching percentage by individual DOAC was calculated as patients switching treatment from the individual DOAC divided by the total number of patients treated with that individual DOAC *100.
- The discontinuation rate was calculated as patients discontinuing treatment divided by patients initiating treatment *100 at 12 months and during the whole study period.

### Sensitivity Analysis

- A sensitivity analysis was performed where patients with DOAC indications other than NVAF recorded in a ± 3-months period around the index date were excluded from the study population.
- For the percentage of overall DOAC discontinuers at 12 months, a sensitivity analysis was performed taking into account a gap of 60 days.

## Results

Between January 1, 2008 and December 31, 2015, 186,405 patients with a diagnosis of NVAF initiated treatment with DOACs in the participating databases (163,017 patients had NVAF diagnosis only, with no other registered diagnosis for DOAC indications in their medical record), 63,542 (34%) of the patients had previous use of VKA (Supplementary Table S3). Of the total number of patients with a diagnosis of NVAF, 91,804 (49.2%), 52,495 (28.2%), and 42,106 (22.6%) received rivaroxaban, dabigatran, and apixaban, respectively (for the number of users in each database see Supplementary Table S4). The cohort characteristics, comorbidities, and co-treatments have been described elsewhere (17).

### Primary Non-Adherence

Primary non-adherence of all DOACs was 11.1% in the BIFAP database (2020 prescriptions (*p*) and 1796 dispensations (*d*)) and 14.0% in the SIDIAP database (13,906 *p*; 11,962 *d*). Primary non-adherence was 8.6% (557 *p*; 506 *d*) and 17.9% (3,321 *p*; 2,728 *d*) for apixaban, 11.4% (492 *p*; 435 *d*) and 11.6% (5,399 *p*; 4,784 *d*) for dabigatran, and 11.9% (971 *p*; 855 *d*) and 14.2% (5,186 *p*; 4,450 *d*) for rivaroxaban in the BIFAP and SIDIAP databases, respectively.

### Persistence Rate

The highest persistence rate for a DOAC at 12 months from treatment initiation was seen for apixaban in the CPRD database (81%) and the lowest rate was seen for dabigatran in the Mondriaan database (22%).

**FIGURE 1:**
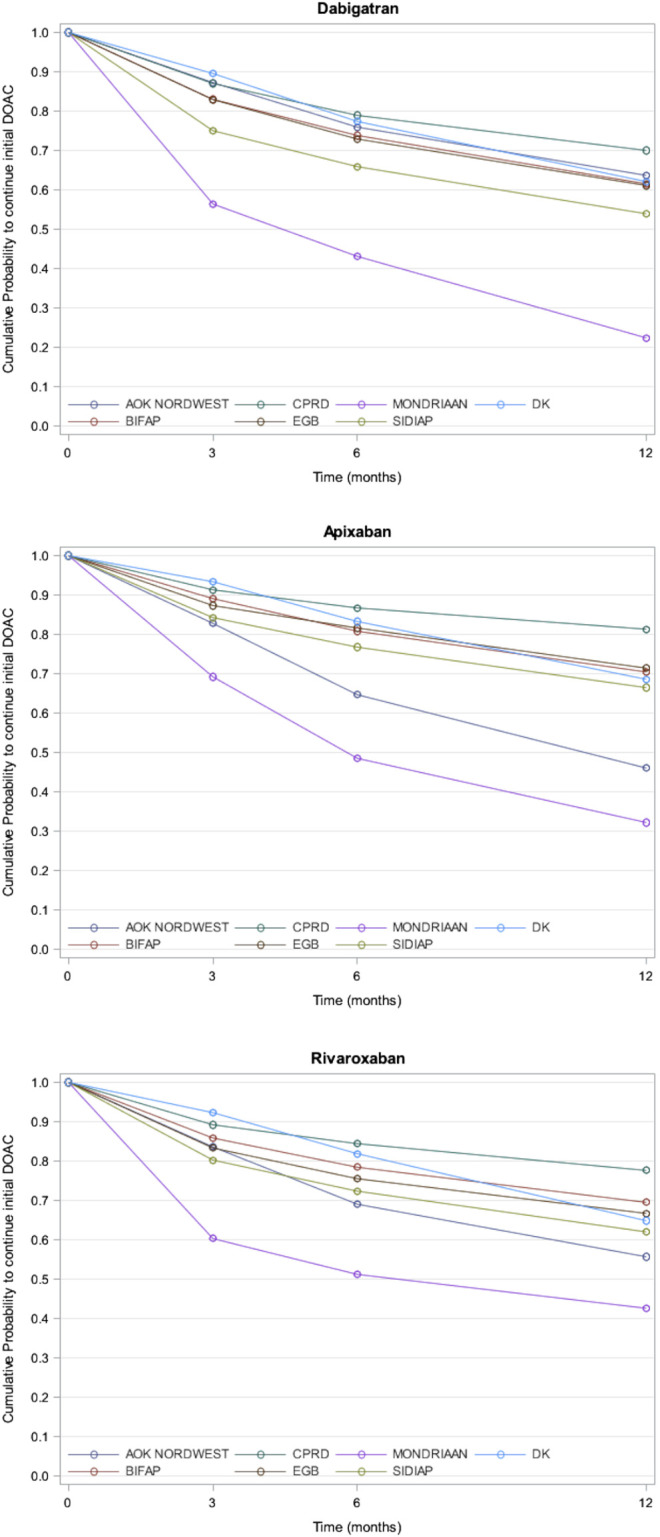
Probability of Continuation of individual DOAC in each database.

Apixaban had the highest persistence at 12 months in all databases, except in the Mondriaan and AOK NORDWEST databases (range: 66% in the SIDIAP database to 81% in the CPRD database). Dabigatran showed the lowest persistence at 12 months in all databases except in the AOK NORDWEST database (range: 22% in the Mondriaan database to 70% in the CPRD database). The crude differences in persistence between each individual DOAC were statistically significant (log-rank test *p* values <0.05) in all databases (Table 2; Figure 1).

**TABLE 2:** Persistence rates at 3, 6, and 12 months by individual DOACs in each database.

		Netherlands (Mondriaan)						Germany (AOK NORDWEST)						Germany (Bavarian claims)					Spain (BIFAP)			
		Persistence (%)		CI 95%		Number of discontinuers/number of remaining patients		Persistence (%)		CI 95%		Number of discontinuers/number of remaining patients		Persistence (%)		CI 95%	Number of discontinuers/number of remaining patients		Persistence (%)	CI 95%		Number of discontinuers/number of remaining patients
**Dabigatran**																						
**3m**		56		(46–67)		40/49		87		(86–88)		495/3,370		NA					83	(82–84)		603/2,668
**6m**		43		(33–53)		51/32		76		(75–77)		929/2,879		NA					74	(72–75)		885/2,144
**12m**		22		(13 -0.32)		64/13		64		(62 -0.65)		1,383/2,306		NA					62	(60–63)		1,217/1,518
**Apixaban**																						
**3m**		69		(57 -0.81)		18/32		83		(82–84)		797/3,717		NA					89	(88–90)		357/2,500
**6m**		49		(34 -0.63)		27/19		65		(63–66)		1,542/2,429		NA					81	(79–82)		571/1884
**12m**		32		(16–49)		31/6		46		(44–48)		2,138/1,158		NA					70	(69–72)		764/939
**Rivaroxaban**																						
**3m**		60		(54–66)		106/145		84		(83–84)		1920/9,700		NA					86	(85–87)		849/4,576
**6m**		51		(45–57)		126/99		69		(68–70)		3,566/7,559		NA					78	(77–79)		1,224/3,727
**12m**		43		(36 -0.49)		140/49		56		(55–57)		4,947/5,266		NA					69	(68 -0.71)		1,600/2,506
**Log-rank test**		pending						<0.001											<0.001			

	Spain (SIDIAP)						United Kingdom (CPRD)						France (EGB)					Denmark (DNR)				
	Persistence (%)		CI 95%		Number of discontinuers/number of remaining patients		Persistence (%)		CI 95%		Number of discontinuers/number of remaining patients		Persistence (%)		CI 95%		Number of discontinuers/number of remaining patients	Persistence (%)			CI 95%	Number of discontinuers/number left
**Dabigatran**																						
**3m**	75		(74–76)		1,174/3,402		87		(85–89)		147/910		83		(79–86)		73/333	89			(89–90)	2,335/18,929
**6m**	66		(64–67)		1,574/2,838		79		(76–81)		226/705		73		(68–77)		111/265	77			(77–78)	4,797/14,909
**12m**	54		(52–55)		2053/1982		70		(67–73)		295/463		61		(56–66)		151/187	62			(61–0.63)	7,559/10,488
**Apixaban**																						
**3m**	84		(83–86)		409/1984		91		(90–93)		152/1,340		87		(83–90)		44/270	93			(93–94)	576/7,640
**6m**	77		(75–78)		566/1,531		87		(85–88)		211/943		82		(77–86)		59/187	83			(82–0.84)	139/5,452
**12m**	66		(64–69)		728/816		81		(79–84)		254/431		71		(65–77)		75/63	68			(67–0.70)	2079/2,491
**Rivaroxaban**																						
**3m**	80		(79–81)		850/3,213		89		(88–90)		339/2,438		83		(81–85)		173/798	92			(92–93)	767/8,416
**6m**	72		(71–74)		1,141/2,587		84		(83–86)		454/1716		76		(73–78)		244/647	82			(81–83)	1,618/6,023
**12m**	62		(60–64)		1,453/1,634		78		(76–79)		561/856		67		(64–70)		312/451	64			(64–66)	2,678/3,338
**Log-rank test**	<0.001						<0.001						0.003					<0.001				

**FIGURE 2:**
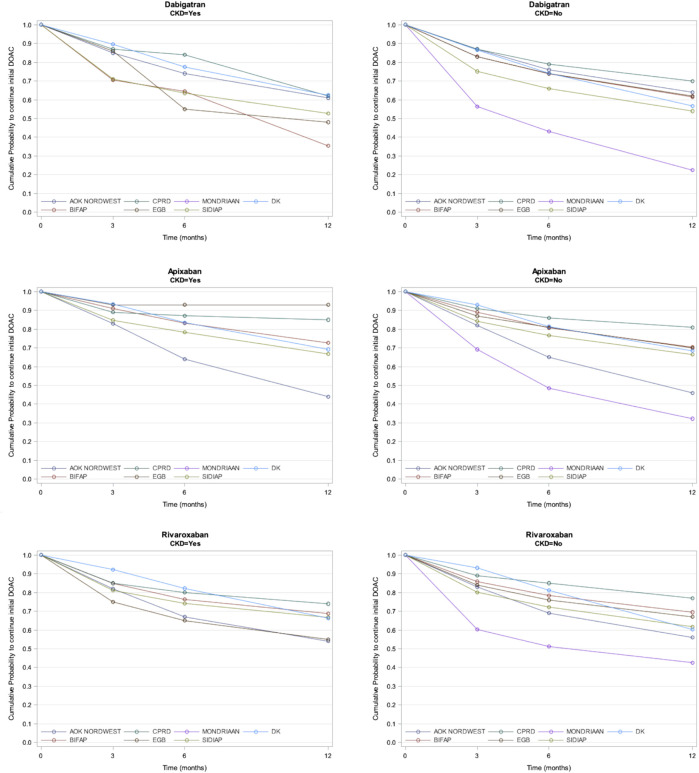
Probability of Continuation of individual DOAC in each database and CKD.

The results regarding persistence by the presence of CKD at baseline by an individual DOAC showed that patients with CKD had a lower persistence to DOACs than those without CKD (Figure 2). Results are not available for the Mondriaan database since no patients with CKD were registered.

### Switching Rate

The cumulative switching percentage at 12 months for all DOAC users ranged from 2.4% in the Mondriaan database to 13.1% in the EGB database. This percentage for the individual DOACs ranged from 1.4% for rivaroxaban in the Mondriaan database to 20% for dabigatran in the EGB database (Table 3). Dabigatran had the highest switching rate at 12 months in all databases. No differences in the switcher rates in the overall period were observed among the different age groups, either for all DOACs or by individual DOACs (Supplementary Table S5).

**TABLE 3:** Switchers and discontinuers: all DOAC, by individual DOAC (at 3, 6, 12 months).

	Netherlands (Mondriaan)	Denmark (DNR)	Germany (AOK NORDWEST)	Germany (Bavarian claims)^a^	Spain (BIFAP)	Spain (SIDIAP)	United Kingdom (CPRD)	France (EGB)
All DOACs	460	44,876	21,718	84,276	14,161	11,962	6,931	2021
Number of **switchers** related to any DOACs N^º^ (%)	11 (2.4%)	5,665 (12.6%)	3,491 (16.1%)	11,791 (14.0%)	1,249 (8.8%)	753 (6.3%)	457 (6.6%)	312 (15.4%)
Switchers at 1–3 months N^º^ (%)	10 (2.2%)	2,914 (6.5%)	1,217 (5.6%)	6,176 (7.3%)	859 (6.1%)	393 (3.3%)	294 (4.2%)	172 (8.5%)
Switchers at 1–6 months N^º^ (%)	10 (2.2%)	3,906 (8.7%)	1827 (8.4%)	7,551 (8.9%)	1,000 (7.1%)	485 (4.0%)	363 (5.2%)	219 (10.8%)
Switchers at 1–12 months N^º^ (%)	11 (2.4%)	4,734 (10.5%)	2,455 (11.3%)	9,409 (11.2%)	1,121 (7.9%)	606 (5.1%)	417 (6.0%)	263 (13.1%)
Number of **discontinuers** related to any DOACs N^º^ (%)	241 (52.4%)	17,795 (39.6%)	11,170 (51.4%)	66,905 (79.4%)	4,540 (32.1%)	5,451 (45.6%)	1,228 (17.7%)	621 (30.7%)
Discontinuers at 1–3 months N^º^ (%)	165 (35.9%)	3,678 (8.2%)	3,158 (14.5%)	32,859 (39.0%)	1809 (12.8%)	2,433 (20.3%)	638 (9.2%)	290 (14.3%)
Discontinuers at 1–6 months N^º^ (%)	203 (44.1%)	7,731 (12.2%)	5,961 (27.4%)	41,785 (49.6%)	2,680 (18.9%)	3,281 (27.4%)	888 (12.8%)	414 (20.5%)
Discontinuers at 1–12 months N^º^ (%)	235 (51.1%)	12,316 (27.4%)	8,435 (38.8%)	53,857 (63.9%)	3,612 (25.5%)	4,234 (35.4%)	1,109 (16.0%)	538 (26.6%)
**Dabigatran**	**99**	**23,308**	**3,968**	**14,729**	**3,863**	**4,784**	**1,265**	**479**
Number of **switchers** related to dabigatran N^º^ (%)	5 (5.1%)	3,942 (16.9%)	1,126 (28.4%)	3,923 (26.6%)	488 (12.6%)	457 (9.5%)	183 (14.5%)	120 (25.0%)
Switchers at 1–3 months N^º^ (%)	4 (4.0%)	1854 (7.9%)	351 (8.8%)	1886 (12.8%)	310 (8.0%)	218 (4.5%)	105 (8.3%)	54 (11.3%)
Switchers at 1–6 months N^º^ (%)	4 (4.0%)	2,452 (10.9%)	531 (13.4%)	2,347 (15.9%)	371 (9.6%)	272 (5.7%)	139 (11.0%)	72 (15.0%)
Switchers at 1–12 months N^º^ (%)	5 (5.0%)	3,158 (13.5%)	708 (17.8%)	2,934 (19.9%)	422 (10.9%)	352 (7.3%)	161 (12.7%)	96 (20.0%)
Number of **discontinuers** related to Dabigatran N^º^ (%)	67 (67.7%)	11,688 (50.1%)	2,387 (60,2%)	10,497 (71.3%)	1,615 (41.8%)	2,798 (58,5%)	353 (27.9%)	184 (38.4%)
Mean treatment duration days (SD)	179.2 (224.2)				397.1 (415.2)	443.1 (438.4)	348.2 (328.5)	360.4 (337.5)
Discontinuers at 1–3 months N^º^ (%)	40 (40.4%)	2,335 (10.1%)	485 (12.2%)	3,850 (26.1%)	603 (16.0%)	1,174 (24.5%)	146 (11.5%)	73 (15.2%)
Discontinuers at 1–6 months N^º^ (%)	50 (50.5%)	4,794 (20.6%)	921 (23.2%)	4,739 (32.2%)	885 (23.0%)	1,574 (32.9%)	225 (17.8%)	111 (23.2%)
Discontinuers at 1–12 months N^º^ (%)	64 (64.6%)	7,559 (32.4%)	1,368 (34.5%)	6,172 (41.9%)	1,225 (31.7%)	2053 (42.9%)	294 (23.2%)	151 (31.5%)
**Apixaban**	**72**	**10,358**	**5,460**	**17,339**	**3,693**	**2,728**	**2060**	**396**
Number of **switchers** related to apixaban N^º^ (%)	2 (2.8%)	540 (5.2%)	366 (6.7%)	1,014 (5.8%)	197 (5.3%)	75 (2.7%)	58 (2.8%)	27 (6.8%)
Switchers at 1–3 months N^º^ (%)	2 (2.8%)	346 (3.3%)	201 (3.7%)	706 (4.1%)	148 (4.0%)	47 (1.7%)	41 (2.0%)	22 (5.6%)
Switchers at 1–6 months N^º^ (%)	2 (2.8%)	436 (4.2%)	266 (4.9%)	835 (4.8%)	170 (4.6%)	57 (2.1%)	48 (2.3%)	27 (6.8%)
Switchers at 1–12 months N^º^ (%)	2 (2.8%)	514 (5.0%)	337 (6.2%)	970 (5.9%)	191 (5.2%)	67 (2.4%)	54 (2.6%)	27 (6.8%)
Number of **discontinuers** related to Apixaban N^º^ (%)	31 (12.9%)	2,475 (23.9%)	2,468 (45.2%)	13,691 (79.0%)	846 (23.0%)	848 (31.1%)	269 (13.1%)	76 (19.2%)
Mean treatment duration days (SD)	118.9 (139.6)				236.8 (203.2)	266.3 (214.7)	217.2 (193.9)	199.8 (157.1)
Discontinuers at 1–3 months N^º^ (%)	19 (26.4%)	576 (5.6%)	781 (14.3%)	7,410 (42.7%)	357 (10.0%)	409 (15.0%)	152 (7.4%)	44 (11.1%)
Discontinuers at 1–6 months N^º^ (%)	27 (37.5%)	1,319 (12.7%)	1,525 (27.9%)	9,587 (55.3%)	571 (15.5%)	566 (20,7%)	210 (10.2%)	59 (14.9%)
Discontinuers at 1–12 months N^º^ (%)	31 (43.1%)	2079 (20.1%)	2,132 (39.0%)	12,060 (69.5%)	770 (20.8%)	728 (26.7%)	254 (12.3%)	75 (18.9%)
	Netherlands (Mondriaan)	Denmark (NRD)	Germany (AOK NORDWEST)	Germany (Bavarian claims)^a^	Spain (BIFAP)	Spain (SIDIAP)	United Kingdom (CPRD)	France (EGB)
Rivaroxaban	289	11,210	12,290	52,208	6,605	4,450	3,606	1,146
Number of **switchers** related to rivaroxaban N^º^ (%)	4 (1.4%)	1,183 (10.5%)	1999 (16.3%)	6,854 (13.1%)	564 (8.5%)	221 (5.0%)	216 (6.0%)	165 (14.4%)
Switchers at 1–3 months N^º^ (%)	3 (1.1%)	714 (6.4%)	665 (5.4%)	3,612 (6.9%)	401 (6.1%)	128 (2.9%)	148 (4.1%)	96 (8.3%)
Switchers at 1–6 months N^º^ (%)	3 (1.1%)	928 (8.3%)	1,030 (8.4%)	4,369 (8.3%)	459 (7.0%)	156 (3.5%)	176 (4.9%)	120 (10.5%)
Switchers at 1–12 months N^º^ (%)	4 (1.4%)	1,062 (9.5%)	1,410 (11.5%)	5,505 (10.5%)	508 (7.7%)	187 (4.2%)	202 (5.6%)	140 (12.2%)
Number of **discontinuers** related to any rivaroxaban N^º^ (%)	143 (51.3%)	3,632 (32.4%)	6,315 (51,4%)	42,717 (81.8%)	2079 (31.5%)	1805 (40.5%)	606 (16.8%)	361 (31.5%)
Mean treatment duration days (SD)	177.7 (199.2)				342.1 (322.4)	339.2 (313.9)	246.7 (235.8)	362.5 (333.2)
Discontinuers at 1–3 months N^º^ (%)	106 (38.0%)	767 (6.8%)	1892 (15.4%)	21,599 (41.4%)	849 (13.0%)	850 (19.1%)	340 (9.4%)	173 (15.1%)
Discontinuers at 1–6 months N^º^ (%)	126 (45.2%)	1,618 (14.4%)	3,515 (28.6%)	27,459 (52.6%)	1,224 (19.0%)	1,141 (25.6%)	453 (12.6%)	244 (21.3%)
Discontinuers at 1–12 months No (%)	140 (50.2%)	2,678 (23.9%)	4,935 (40.2%)	35,625 (68.2%)	1,617 (24.0%)	1,453 (32.6%)	561 (15.5%)	312 (27.2%)

a In the Bavarian databases due to the quarterly definition, an interruption of one quarter could not necessarily mean a termination of the therapy in all cases.

### Discontinuation Rate

Cumulative discontinuation rates of DOACs at 12 months ranged from 16% in the CPRD database to 63.9% in the Bavarian CD database. The cumulative discontinuation percentage at 12 months ranged from 12.3% for apixaban in the CPRD database to 69.5% for apixaban in the Bavarian CD database (Table 3).

Dabigatran had the highest percentage of discontinuers at 12 months in all databases (from 23.2% in the CPRD database to 64.6% in the Mondriaan database), except in the Bavarian CD and AOK NORDWEST databases, where apixaban and rivaroxaban had the highest proportion of discontinuers (69.5 and 40.2%, respectively) (Table 3).

No differences were observed among the different age groups regarding discontinuation rates during the whole study period, either overall or by individual type of DOAC. The only exception was in the 75–79 years age group in the Mondriaan database, where the percentage of discontinuers was very low (10%) compared to <75 years (61.6%) and ≥80 years (70%) (Supplementary Table S5).

### Sensitivity Analysis

The analysis performed in users that received DOACs indicated for treating NVAF and had no other DOAC registered indications did not differ from the main analysis for switchers and discontinuers (Supplementary Table S6).

The analysis where a 60-days gap was applied to construct treatment episodes resulted in a decrease of discontinuation percentage for all patients compared to the main analysis, regardless of the type of DOAC used. The overall rates of DOAC discontinuers at 12 months decreased, ranging from 10.2% in the CPRD database to 37.0% in the Mondriaan database (for detailed information on the sensitivity analysis see Supplementary Tables S6–S8 of the Supplementary Material).

## Discussion

This study provides an overview of DOAC adherence in eight different databases from six European countries. To the best of our knowledge, this is the first study displaying information on primary non-adherence and persistence to DOAC treatment in several European countries at a national or regional level using a common protocol approach.

Primary non-adherence of all DOACs was 11.1% in the BIFAP database and 14.0% in the SIDIAP database. Apixaban is the DOAC with the highest persistence rate and lowest discontinuation rate at 12 months in all databases except in the German AOK and Dutch Mondriaan databases. Dabigatran had the highest proportion of discontinuers and switchers at 12 months in most databases.

### Primary Non-Adherence

Our results on primary non-adherence of all DOACs comparing two Spanish databases covering different populations are quite similar to those described in a study performed in the Valencia region of Spain, with primary non-adherence seen in 11% of all patients that received a prescription for a DOAC in the BIFAP database and 14% in the SIDIAP database (Rodriguez-Bernal et al., 2018). However, differences were found among individual DOACs between both databases. Apixaban had the lowest percentage of primary non-adherence (9%) in the BIFAP database while it showed the highest percentage (18%) in the SIDIAP database. The study from Valencia showed high differences between individual DOACs (from 5% for apixaban to 16% for rivaroxaban) (Rodriguez-Bernal et al., 2018). The progressive and lowest implementation, from 2011 in BIFAP, of the possibility to identify the dispensation linked to the prescription could explain the differences between BIFAP and SIDIAP.

Polypharmacy, co-payment, and age were inversely related to higher primary non-adherence in the study from Valencia (Rodriguez-Bernal et al., 2018). A specific sub-analysis of our data showed several factors that may influence primary non-adherence, including which DOAC is prescribed, age, and diagnosis of CKD, diabetes, hypertension, or stroke/TIA (Charlton et al., 2021).

### Persistence Rates

Our results on persistence rates results are in accordance with what has been shown in other studies (Nelson et al., 2015; Coleman et al., 2016; ; Mueller et al., 2017). The inclusion criteria in those studies are similar to ours, even though that the gap chosen after the end of supply to consider a drug to be discontinued was wider than ours (90 vs 30 days) in both studies (Nelson et al., 2015; Coleman et al., 2016).

The observed lower persistence in patients initiating dabigatran use when compared to patients initiating other DOACs might be due to reported frequent adverse events (e.g., dyspepsia or bleeding complications) (Nelson et al., 2015; Alberts et al., 2016). The need of more frequent dosing for dabigatran and apixaban compared to rivaroxaban may explain some differences in the persistence (Nelson et al., 2015; McHorney et al., 2017).

In a published meta-analysis of 36 observational studies the overall pooled proportion of persistence for all follow-up duration was 69% for all DOACs, 74% for apixaban, 62% for dabigatran, and 72% for rivaroxaban. These results are in accordance with our study as apixaban presented the highest persistence rate at 12 months and dabigatran the lowest (Ozaki et al., 2020).

### Switching Rates

In other published studies, the switching percentage was higher for dabigatran (17.0–24.7%) compared to rivaroxaban (14.3–17.6%) (Hellfritzsch et al., 2017; Maura et al., 2017), which is consistent with our results. Moreover, the special precaution or contraindication of use of DOACs in patients with different levels of renal impairment could also help explain either the switch in some cases or the discontinuation in others. In addition, the ARISTOTLE trial publication showed that apixaban had less risk of bleeding than warfarin which could have favored the higher switching to apixaban (Granger et al., 2011).

The switching percentages at 12 months for each one of the DOACs in the EGB, DNR, and CPRD databases in our study are slightly different (ranged from 2.6 to 20%) than those observed in other studies performed in France, Denmark, and the United Kingdom (Durham et al., 2017; Hellfritzsch et al., 2017; Maura et al., 2017) (ranged from 24.7 to 7%). In the French study, the switchers were defined as patients who had at least one reimbursement for VKA or for a DOAC that was different from the one initially received. This definition was different from our study (Maura et al., 2017). In the Danish study, using the Danish National Prescription Registry (Hellfritzsch et al., 2017), the different exclusion criteria (excluding those patients with a history of valvular atrial fibrillation) in our study may explain the difference. In the published study using the CPRD database the inclusion criteria were different as they included new oral anticoagulants and DOAC users (Durham et al., 2017). In these three studies, as well as in ours, dabigatran was the DOAC with the highest percentage of switchers, maybe because it was poorly tolerated in some patients (Connolly et al., 2009).

An observational retrospective Dutch study using pharmacy dispensing data from the Foundation of Pharmaceutical Statistics (SFK) showed that 31% of incident DOAC users with atrial fibrillation (valvular atrial fibrillation included) switched to another anticoagulant at 12 months, with the majority switching to a VKA (67%), while 34% had discontinued at 12 months (Zielinski et al., 2019). In our study, the Mondriaan database showed a lower switching percentage and had the lowest switching percentages compared to the other databases. Perhaps the exclusion of the valvular atrial fibrillation, the lowest population coverage and the inclusion of specialist dispensing in the SFK may explain these differences (Smeets et al., 2018).

### Discontinuation Rate

Regarding the discontinuation percentages, the information that has emerged from the pivotal clinical trials is diverse. The discontinuation percentage at 12 months was 15.5% for dabigatran (Connolly et al., 2009). For apixaban, the discontinuation percentage during the study period was 25.3% (34) and the percentage of stopped treatments during the study period for rivaroxaban was 23.7% (Patel et al., 2011). We have to mention that the exclusion criteria in these clinical trials were wider than those in our study.

Other studies performed in Germany and Denmark (regional and nationwide registries, respectively) (Beyer-Westendorf et al., 2015; Hellfritzsch et al., 2017) showed discontinuation rates at 12 months of 15% for new users of rivaroxaban in the German study and 15.5% of dabigatran users to 11.4% of apixaban users in the Danish study. Both studies included patients with atrial fibrillation without excluding those with diagnoses of cardiac valvular disease, and both included naive patients and patients switching from VKA. However, the German study may reflect a selected population of moderate-high risk patients, which may explain the observed differences. In the Danish study, the allowed gap for discontinuation was 60 days, which is similar to the one used in our sensitivity analysis. As expected, the results of our sensitivity analysis for the Danish database decreased the percentage of discontinuers and were more similar to those of the referenced studies. The different proportion of patients with previous use of VKA included in the German and Danish studies (Beyer-Westendorf et al., 2015; Hellfritzsch et al., 2017) and the differences in the inclusion criteria might explain some of the divergences. In addition in the Bavarian databases due to the quarterly definition, an interruption of one quarter could not necessarily mean a termination of the therapy in all cases (e.g., two prescriptions were prescribed in one quarter and then a quarter was skipped).

Bleeding complications and the changes in underlying disease severity (e.g., restoration of sinus rhythm) have been found to be the main reason for DOAC discontinuation (Beyer-Westendorf et al., 2015; Hellfritzsch et al., 2017). Unfortunately, these data are not available in our study, but the high proportion of discontinuers warns us that we must be aware of these cases in clinical practice.

The different approval dates for each DOAC (dabigatran was the first DOAC to be approved and apixaban is the more recent) could play a role in interpreting these results since older drugs might have a higher probability of being either switched or discontinued to new ones (Ibáñez et al., 2019). Moreover the variability in approval dates for each DOAC can translate into patterns of use in the post-approval period that may vary over time (Nelson et al., 2015).

Knowledge on the clinical consequences of either switching or discontinuation of DOACs has been estimated in a post hoc analysis of the pivotal trials in which it was associated with an increase in bleeding and thromboembolism (Patel et al., 2013; Granger et al., 2015). Furthermore, in a Swedish observational study non-persistence and poor adherence were both associated with increased stroke risk [non-persistence adjusted odds ratio (aOR): 2.05 (Komen et al., 2020).

### Strengths and Limitations

The main strength is the use of eight databases that provided a large amount of real-world adherence data about patients receiving new DOAC prescriptions in six European countries.

Common protocol and data specifications were used by all participants, with consistent inclusion and exclusion criteria for users, operational case definitions, and common analytical procedures in order to minimize methodological discrepancies as much as possible and facilitate comparison of results across data sources. In addition, results were blinded and shared with the whole consortium only after each center had completed their analysis, avoiding some information bias and promoting independent results. Moreover, the results of the sensitivity analysis excluding the users with other indications than NVAF were not different from the main analysis for switchers and discontinuers. This suggests that the analysis with the whole included population is representative of DOAC use for NVAF. Primary non-adherence has been shown to predict health outcomes (Jackevicius et al., 2017; Rodriguez-Bernal et al., 2018). Therefore it is important to have information about primary non-adherence. Our results could be useful as an approximation of the percentage of non-dispensed prescriptions when databases have only dispensing data (Rodriguez-Bernal et al., 2018). Unfortunately, in the present study, this information was only available for two databases (BIFAP and SIDIAP).

In terms of limitations, we should first mention that validation of adherence to the first prescription, discontinuation, or switching through confirmation by physicians, patients, or clinical history notes was not performed. It could happen that follow-up censoring precluded us from knowing the real continuation of the patient treatment in other institutions (e.g., hospitals, nursing homes). Secondly, first prescriptions issued in the specialist setting were not included for Spain, the Netherlands, and the United Kingdom, leading to a potential underestimation of the real-time lapsed from treatment initiation.

The assumption of the permissible days between prescriptions and reimbursements to assume continuity (30-days gap) could have affected the estimation of discontinuation and switching proportions (Simons et al., 2016). In the sensitivity analysis, taking into account a permissible gap of 60 days, the discontinuation percentage decreased for all DOACs and each individual drug. Overlapping prescriptions or dispensations were not taken into account which could also have affected these measures.

In addition, inherent differences in the coding systems used in the databases may also have created differences in capturing the diagnosis of NVAF although efforts have been made to unify the different code systems. Despite the fact that codes had not been validated in this study, outcome validation has been performed in other studies showing high validity (Ibáñez et al., 2019). Information on the indication associated with the prescription might be incomplete since definite linkage between compound and indication is lacking in most of the databases (Ibáñez et al., 2019).

Differences in the database characteristics such as population and drug coverage (DNR is the only national database), prescription or dispensing databases, and reimbursement conditions over time could partly explain the observed differences among databases (Ibáñez et al., 2019). Additionally, each country has different health policies, national prescribing guidelines, and prescription patterns, which, among other intrinsic characteristics of the populations and their lifestyle, may contribute to the variability of results among the databases (Komen et al., 2017; Vlahović-Palcevski et al., 2016).

Our results suggest that adherence multifaceted interventions such as counseling or daily treatment support are needed and should be systematically encouraged at the DOAC initiation and repeatedly throughout the course of therapy (Vrijens B, 2016), although evidence of the interventions effects are inconsistent from study to study, and only a minority of controlled studies show an improvement on both adherence and clinical outcomes. Adherence is acknowledged as a complex behavior. Current methods of improving medication adherence for chronic health problems are mostly complex and not very effective, so that the full benefits of treatment cannot be realized (Nieuwlaat et al., 2014). There is a need for further research and education to improve future DOAC utilization.

This is a cross-national comparison study using real-world longitudinal data collected in eight European electronic health care databases.

There were differences in the adherence characteristics and individual DOACs. The variations among database results might be explained by their characteristics, differences in national guidelines prescription pattern diversity, and the demographic characteristics of the population included. However, the overall adherence findings throughout the databases point to a common direction with a consistent similar pattern.

The high discontinuation percentage observed in several countries (more than 50%) and the persistence rates varying from 22 to 81% between centers will require a detailed analysis of reasons and consequences of the prophylaxis of cerebrovascular events in patients with NVAF and whether DOAC treatment results in better outcomes.

## Data Availability

The data analyzed in this study was obtained from the following databases: Julius General Practitioner Network (JHN), Danish National Registries (DNR), AOK NORDWEST database, Bavarian Association of Statutory Health Insurance Physicians database, base de datos para la Investigación Farmacoepidemiológica en Atención Primaria (BIFAP), Information System for Development of Research in Primary Care (SIDIAP), Clinical Practice Research Datalink (CPRD), Echantillon Généraliste de Bénéficiaires (EGB). Information on whom to contact with requests to access these datasets should be directed to EUPEPV@uu.nl.
