# Supplementary material for: Adherence to Direct Oral Anticoagulants in Patients With Non-Valvular Atrial Fibrillation: A Cross-National Comparison in Six European Countries (2008–2015)
Source: Front Pharmacol. 2021 Nov 3;12:682890. doi: 10.3389/fphar.2021.682890 (PMC8596153; doi:10.3389/fphar.2021.682890)
Supplement: Supplementary file 1 [file DataSheet1.pdf]

Table S1. Codes used to identify the study population.

Study population	ICD-10	ICD-8	ICD-9-CM	ICD-10-GM	ICPC
**Diagnosis**					
Atrial fibrillation	I48	427.93, 427.94	427.31 427.32	I48	K78
**Exclusion**					
Valvular atrial fibrillation	I05.0, I05.1, I05.2, I05.8, I05.9, I06, I08, I34, I35 Z95.2, Z95.3, Z95.4	424.0, 424.1, 394, 395, 396 494.0	394.0, 394.1, 394.2, 394.9, 395, 396, 424, V42.2, V43.3	I05.0, I05.1, I05.2, I05.8, I05.9, Z95.2, Z95.3, Z95.4, I06, I08, I34, I35	K71, K83

[a]Patients were considered to have a diagnosis of NVAF according to 1) A diagnosis of NVAF was linked to the first prescriptions of the DOAC (BIFAP, Mondriaan), if this was not possible then 2) a medical code for NVAF ±3 months around the index date either in GP-records (CPRD, SIDIAP, BIFAP, Mondriaan) or claims records (AOK Nordwest, Bavarian CD) database) or 3) a medical code for NVAF prior to index date to 3 months after the index date in case of Hospital-record database (DNR, EGB).

Table S2. Codes used to identify chronic kidney disease.

	ICD-10	ICD-8	ICD-9-CM	ICD-10-GM	ICPC
**Diagnosis**					
Severe renal impairment/Chronic and acute kidney disease	I12, I13, N00, N01, N03, N04, N05, N07, N08, N10, N11, N12, N14, N17, N18.4, N18.5, N18.9 N19, Q61, Z94.0	(Charlson Index) 403; 404; 580-584; 590.09	403.01 403.11 403.91 404.02, 404.03, 404.12, 404.13, 404.92, 404.93, 580.8, 581, 582, 585.3, 584, 585.5, 585.5, 585.6, 585.9, 586, 588, 590.1, 590.20, 590.8, 753.1 V42.0	I12 I13 N00 N01 N03 N04 N05 N07 N08, N10, N11, N12, N14, N17, N18.4, N18.5, N18.9 N19, Q61, Z94.0	U99.01 ICPC codes extension in BIFAP[i] U99.10-CHRONIC KIDNEY DISEASE U99.9-ACUTE KIDNEY DISEASE

Table S3. Baseline characteristics for all new users of direct oral anticoagulants stratified by prior use of oral anticoagulants by database.

	MONDRIAAN	DANISH NATIONAL REGISTRIES	AOK NORDWEST	BAVARIAN CLAIMS	BIFAP	SIDIAP	CPRD	EGB
**ALL USERS**	460	44876	21718	84276	14161	11962	6931	2021
**WITHOUT PREVIOUS USE OF OACs**	**303 (65.9%)**	**31931 (71.1%)**	**13509 (62.2%)**	**55515 (65.9%)**	**8807 (62.2%)**	**5630 (47.1%)**	**5712 (82.4%)**	**1456 (72.0%)**
**Female**	127 (41.9%)	14451 (45.3%)	7184 (53.2%)	28551 (51.4%)	4100 (46.6%)	2462 (43.7%)	2526 (44.2%)	630 (43.3%)
**Male**	176 (58.1%)	17480 (54.7%)	6325 (46.8%)	26964 (48.6%)	4707 (53.4%)	3168 (56.3%)	3186 (55.8%)	826 (56.7%)
**AGE in years, mean (SD)**	69.9 (11.6)	73.3 (11.2)	73.9 (11.8)	73.4 (11.5)	75.4 (10.8)	73 (12)	74.8 (11.0)	72.1 (12.3)
**CO-MORBIDITIES**	-	19883 (62.3%)	11251 (83.3%)	46289 (83.4%)	3718 (42.2%)	2476 (44.0%)	3458 (60.5%)	677 (46.5%)
**CONCOMITANT MEDICATION**	0 (0.0%)	12089 (37.9%)	1426 (10.6%)	20881 (37.6%)	1192 (13.5%)	461 (8.2%)	437 (7.7%)	602 (41.3%)
**WITH PREVIOUS USE OF OACs**	**157 (34.1%)**	**12945 (28.8%)**	**8209 (37.8%)**	**28761 (34.1%)**	**5354 (37.8%)**	**6332 (52.9%)**	**1219 (17.6%)**	**565 (28%)**
**Female**	62 (39.5%)	5880 (45.4%)	4466 (54.4%)	15519 (54.0%)	2655 (49.6%)	3136 (49.5%)	523 (42.9%)	237 (41.9%)
**Male**	95 (60.5%)	7065 (54.6%)	3743 (45.6%)	13242 (46.0%)	2699 (50.4%)	3196 (50.5%)	696 (57.1%)	328 (58.1%)
**AGE in years, mean (SD)**	70.1 (10.5)	75.2 (10.1)	76.4 (9.9)	76.7 (9.6)	76.3 (9.8)	76.0 (9.7)	74.3 (10.9)	74.6 (11.0)
**CO-MORBIDITIES**	-	10076 (77.8%)	7674 (93.4%)	26999 (93.9%)	3147 (58.8)	3961 (62.6%)	879 (72.1%)	365 (64.6%)
**CONCOMITANT MEDICATION**	0 (0.0%)	5367 (41.5%)	658 (8.0%)	13749 (47.8%)	641 (12.0%)	181 (2.9%)	88 (7.2%)	115 (20.3%)

Table S4. Number of new users for all DOACs and each type of DOAC by database

	MONDRIAAN		DANISH NATIONAL REGISTRIES		AOK NORDWEST		BAVARIAN CLAIMS		BIFAP		SIDIAP		CPRD		EGB	
	N	(%)	N	(%)	N	(%)	N	(%)	N	(%)	N	(%)	N	(%)	N	(%)
**All DOACs**	460	(100)	44876	(100)	21718	(100)	84276	(100)	14161	(100)	11962	(100)	6931	(100)	2021	(100)
Dabigatran	99	(21,5)	23308	(51.9)	3968	(18.3)	14729	(17.4)	3863	(27.3)	4784	(40.0)	1265	(18.3)	479	(23.7)
Apixaban	72	(15,7)	10358	(23.1)	5460	(25.1)	17339	(20.6)	3693	(26.1)	2728	(22.8)	2060	(29.7)	396	(19.6)
Rivaroxaban	289	(62,8)	11210	(25.0)	12290	(56.6)	52208	(61.9)	6605	(46.6)	4450	(37.2)	3606	(52.0)	1146	(56.7)

Table S5. Switchers and discontinuers: all DOAC, by type of DOAC and by sex and age in each database, for the complete study period.

	Netherlands (Mondriaan)	Denmark (DNR)	Germany (AOK NORDWEST)	Germany (Bavarian claims)	Spain (BIFAP)	Spain (SIDIAP)	United Kingdom (CPRD)	France (EGB)
**All DOACs**	**460**	**44876**	**21718**	**84276**	**14161**	**11962**	**6931**	**2021**
SWITCHERS *(all study period)*	11 (2.4%)	5665 (12.6%)	3491 (16.1%)	11791 (14.0%)	1249 (8.8%)	753 (6.3%)	457 (6.6%)	312 (15.4%)
Female	4 (2.1%)	2544 (12.5%)	1860 (16.0%)	6332 (14.4%)	621 (9.2%)	373 (6.7%)	232 (7.6%)	155 (17.9%)
Male	7 (2.6%)	3121 (12.7%)	1631 (16.2%)	5459 (13.6%)	628 (8.5%)	380 (6.0%)	225 (5.8%)	157 (13.6%)
<75 year	7 (2.3%)	3419 (14.9%)	1501 (17.1%)	5297 (14.4%)	540 (9.7%)	336 (6.5%)	204 (6.6%)	131 (13.2%)
75-79 years	3 (4.9%)	879 (12.1%)	830 (17.3%)	2798 (15.6%)	256 (9.7%)	150 (6.7%)	100 (7.7%)	65 (18.4%)
>=80 years	1 (1.0%)	1367 (9.3%)	1160 (14.2%)	3696 (12.5%)	453 (7.6%)	267 (5.9%)	153 (6.1%)	116 (17.2%)
Female <75 year	1 (0.8%)	1260 (15.8%)	606 (17.6%)	2368 (15.6%)	222 (11.2%)	142 (7.9%)	82 (8.1%)	46 (15.4%)
Female 75 -79 years	3 (12.5%)	452 (12.9%)	472 (17.6%)	1582 (16.3%)	138 (10.1%)	74 (6.6%)	58 (9.4%)	31 (20.4%)
Female >=80 years	0 (0.0)	832 (9.4%)	782 (14.2%)	2382 (12.4%)	261 (7.7%)	157 (5.9%)	92 (6.5%)	78 (18.7%)
Male <75 year	8 (4.2%)	2159 (14.4%)	895 (16.7%)	2929 (13.6%)	318 (8.9%)	194 (5.7)	122 (5.8%)	85 (12.2%)
Male 75 -79 years	0 (0.0)	427 (11.4%)	358 (17.1%)	1216 (14.7%)	118 (9.2%)	76 (6.8%)	42 (6.2%)	34 (16.9%)
Male >=80 years	1 (2.3%)	535 (9.2%	378 (14.4%)	1314 (12.6%)	192 (7.5%)	110 (5.9%)	61 (5.5%)	38 (14.8%)
DISCONTINUERS*(all study period)*	241 (52.4%)	17795 (39.6%)	11170 (51.4%)	66905 (79.4%)	4540 (32.1%)	4698 (39.35)	1228 (17.7%)	621 (30.7%)
Female	107 (56.6%)	7606 (37.4%)	5905 (50.7%)	34853 (79.4%)	2099 (31.1%)	2102 (37.5%)	499 (16.4%)	248 (28.6%)
Male	134 (49.4%)	10189 (41.5%)	5265 (52.3%)	32052 (79.7%)	2441 (33.0%)	2596 (40.8%)	729 (18.8%)	373 (32.3%)
<75 year	167 (61.6%)	9640 (41.9%)	4375 (49.7%)	28985 (79.1%)	1817 (32.7%)	2228 (43.1%)	563 (18.1%)	340 (34.2%)
75-79 years	31 (10.0%)	2766 (38.1%)	2331 (47.7%)	13975 (77.7%)	848 (32.0%)	833 (37.0%)	196 (15.1%)	95 (26.9%)
>=80 years	43 (70.5%)	5389 (36.8%)	4464 (54.8%)	23945 (80.8%)	1875 (31.5%)	1637 (36.0%)	469 (18.6%)	186 (27.6%)
Female <75 year	70 (70.7%)	3071 (38.5%)	1636 (47.4%)	11811 (77.9%)	619 (31.1%)	724 (40.4%)	145 (14.3%)	99 (33.1%)
Female 75 -79 years	12 (50.0%)	1292 (36.9%)	1288 (47.9%)	7530 (77.4%)	437 (31.9%)	408 (36.3%)	92 (14.9%)	37 (24.3%)
Female >=80 years	25 (44.6%)	3243 (36.6%)	2981 (54.1%)	15512 (80.8%)	1043 (30.7%)	970 (36.2%)	262 (18.5%)	112 (26.9%)
Male <75 year	97 (50.8%)	6569 (43.8%)	2739 (51.2%)	17174 (79.9%)	1198 (33.6%)	1504 (44.6%)	418 (19.9%)	241 (34.6%)
Male 75 -79 years	19 (51.4%)	1474 (39.2%)	1043 (49.7%)	6445 (78.1%)	411 (32.2%)	425 (37.8%)	104 (15.3%)	58 (28.9%)
Male >=80 years	18 (41.9%)	2146 (37.0%)	1483 (56.5%)	8433 (80.7%)	832 (32.5%)	667 (35.7%)	207 (18.8%)	74 (28.8%)
CKD n [ndiscontinuers (%)]	0	2055 [758 (36.9%)]	4377 [2286(52.2%)]	20287 [15840 (78.1%)]	226 [67 (29.6%)]	1837 [641 (34.9%)]	51 [8 (15.7%)]	107 [39 (36.4%)]

Table S6. Number of New DOACs users, switchers and discontinuers with NVAF excluding those with other approved DOAC indications.

	Netherlands (Mondriaan)	Denmark (NRD)	Germany (AOK NORDWEST)	Germany (Bavarian claims)	Spain (BIFAP)	Spain (SIDIAP)	United Kingdom (CPRD)	France (EGB)
**All DOACs**	**NOT APPLICABLE - NO patients with other indications**	**41708**	**18239**	**70970**	**13719**	**10287**	**6611**	**1483**
Number of **switchers** related to any DOACs Nº (%)		5240 (12.6)	2880 (15.8)	9824 (13.8)	1200 (8.7)	641 (6.2)	428 (6.5)	227 (15.3)
Switchers at 1-3 months Nº (%)		2656 (6.4)	1012 (5.5)	5159 (7.3)	827 (6.0)	344 (3.3)	273 (4.1)	121 (8.2)
Switchers at 1-6 months Nº (%)		3566 (8.4)	1520 (8.3)	6289 (8.9)	959 (7.0)	419 (4.1)	338 (5.1)	153 (10.3)
Switchers at 1-12 months Nº (%)		4350 (10.4)	2024 (11.1)	7815 (11.0)	1077 (7.8)	525 (5.1)	389 (5.9)	188 (12.7)
Number of **discontinuers** related to any DOACs Nº (%)		16523 (39.6)	9441 (51.8)	56478 (79.6)	4392 (32.0)	4096 (39.8)	1155 (17.5)	448 (30.2)
Discontinuers at 1-3 months Nº (%)		3424 (8.2)	2654 (14.5)	27586 (38.9)	1743 (12.7)	1777 (17.3)	592 (9.0)	205 (13.8)
Discontinuers at 1-6 months Nº (%)		7165 (17.2)	5016 (27.5)	35148 (49.5)	2592 (18.9)	2438 (23.7)	831 (12.6)	293 (19.8)
Discontinuers at 1-12 months Nº (%)		11357 (27.2)	7098 (38.9)	45344 (63.9)	3495 (25.5)	3151 (30.6)	1041 (15.7)	385 (26.0)
**Dabigatran**		**21885**	**3405**	**12604**	**3749**	**4200**	**1223**	**358**
Number of **switchers** related to Dabigatran Nº (%)		3672 (16.8)	951 (27.9)	3343 (26.5)	473 (16.6)	394 (9.4)	173 (14.1)	88 (24.6)
Switchers at 1-3 months Nº (%)		1705 (7.8)	300 (8.8)	1581 (12.5)	301 (8.0)	192 (4.6)	97 (7.9)	39 (10.9)
Switchers at 1-6 monthsNº (%)		2339 (10.7)	454 (13.3)	2007 (15.9)	358 (9.5)	235 (5.6)	130 (10.6)	50 (14.0)
Switchers at 1-12 months Nº (%)		2924 (13.4)	595 (17.5)	2501 (19.8)	409 (10.9)	307 (7.3)	152 (12.4)	69 (19.3)
Number of **discontinuers** related to Dabigatran Nº (%)		11000 (50.3)	2064 (60.6)	8992 (71.3)	1564 (41.7)	2087 (49.7)	342 (28.0)	135 (37.7)
Discontinuers at 1-3 months Nº (%)		2197 (10.0)	424 (12.4)	3275 (26.0)	582 (15.5)	850 (20.2)	140 (11.4)	50 (14.0)
Discontinuers at 1-6 months Nº (%)		4503 (20.6)	797 (23.4)	4032 (32.0)	857 (22.9)	1159 (27.6)	219 (17.9)	79 (22.1)
Discontinuers at 1-12 months Nº (%)		7093 (32.4)	1167 (34.3)	5264 (41.8)	1184 (31.6)	1516 (30.1)	285 (23.3)	109 (30.4)
**Apixaban**		**9669**	**4622**	**14503**	**3585**	**2299**	**1967**	**279**
Number of **switchers** related to Apixaban Nº (%)		497 (5.1)	297 (6.4)	827 (5.7)	189 (5.3)	69 (3.0)	54 (2.7)	19 (6.8)
Switchers at 1-3 months Nº (%)		313 (3.2)	163 (3.5)	579 (4.0)	142 (4.0)	45 (1.2)	37 (1.9)	14 (5.0)
Switchers at 1-6 months Nº (%)		398 (4.1)	215 (4.6)	680 (4.7)	162 (4.5)	54 (2.3)	44 (2.2)	19 (6.8)
Switchers at 1-12 months Nº (%)		472 (4.9)	274 (5.9)	794 (5.5)	183 (5.1)	63 (2.7)	50 (2.5)	19 (6.8)
Number of **discontinuers** related to Apixaban Nº (%)		2297 (23.7)	2102 (45.5)	11496 (79.3)	827 (23.1)	636 (27.7)	253 (12.9)	56 (20.1)
Discontinuers at 1-3 months Nº (%)		542 (5.6)	667 (14.4)	6216 (42.8)	349 (9.7)	303 (13.2)	141 (7.2)	36 (12.9)
Discontinuers at 1-6 months Nº (%)		1223 (12.6)	1303 (28.2)	8054 (55.5)	557 (15.5)	426 (18.5)	196 (10.0)	45 (16.1)
Discontinuers at 1-12 months Nº (%)		1917 (19.8)	1817 (39.3)	10124 (69.8)	753 (21.0)	544 (23.7)	238 (12.1)	55 (19.7)

	Netherlands (Mondriaan)	Denmark (NRD)	Germany (AOK NORDWEST)	Germany (Bavarian claims)	Spain (BIFAP)	Spain (SIDIAP)	United Kingdom (CPRD)	France (EGB)
Rivaroxaban		10154	10212	43863	6385	3788	3421	846
Number of switchers related to Ribaroxavan Nº (%)		1071 (10.5)	1632 (16.0)	5654 (12.9)	538 (8.4)	178 (4.7)	201 (5.9)	120 (14.2)
Switchers at 1-3 months Nº (%)		638 (6.3)	549 (5.4)	2999 (6.8)	384 (6.0)	107 (2.8)	139 (4.1)	68 (8.0)
Switchers at 1-6 months Nº (%)		829 (8.2)	851 (8.3)	3602 (8.2)	439 (6.9)	130 (3.4)	164 (4.8)	84 (9.9)
Switchers at 1-12 months Nº (%)		954 (9.4)	1155 (11.3)	4520 (10.3)	485 (7.6)	155 (4.1)	187 (5.5)	100 (11.8)
Number of discontinuers related to any Rivaroxaban Nº (%)		3226 (31.8)	5275 (51.6)	35990 (82.0)	2001 (31.3)	1373 (36.2)	560 (16.4)	257 (30.4)
Discontinuers at 1-3 months Nº (%)		685 (6.7)	1563 (15.3)	18095 (41.2)	812 (17.7)	624 (16.5)	311 (9.1)	119 (14.1)
Discontinuers at 1-6 months Nº (%)		1439 (14.2)	2916 (28.5)	23062 (52.6)	1178 (18.4)	853 (22.5)	416 (12.2)	169 (20.0)
Discontinuers at 1-12 months Nº (%)		2347 (23.1)	4114 (40.3)	29956 (68.3)	1558 (24.4)	1091 (20.8)	518 (15.1)	221 (26.1)

Table S7. Number of New DOACs users with NVAF (sensitivity analyses taking account a gap of 60 days)

	Netherlands (Mondriaan)	Denmark (NRD)	Germany (AOK NORDWEST)	Germany (Bavarian claims)	Spain (BIFAP)	Spain (SIDIAP)	United Kingdom (CPRD)	France (EGB)
**All DOACs**	**460**	**44876**	**NA**	**NA**	**14161**	**11962**	**6931**	**2021**
Number of **discontinuers** related to any DOACs Nº (%)		10620 (23.7)			2816 (20.0)	4081 (31.1)	779 (11.2)	470 (23.3)
Discontinuers at 1-3 months Nº (%)	107 (23.3)	2972 (6.6)			1054 (7.4)	1717 (14.3)	380 (5.5)	221 (10.9)
Discontinuers at 1-6 months Nº (%)	140 (30.4)	5793 (12.9)			1561 (11.0)	2355 (19.7)	549 (7.9)	321 (15.9)
Discontinuers at 1-12 months Nº (%)	170 (37.0)	8374 (18.7)			2135 (15.1)	3034 (25.4)	708 (10.2)	417 (20.6)
**Dabigatran**	**99**	**23308**			**3863**	**4784**	**1265**	**479**
Number of **discontinuers** related to Dabigatran Nº (%)		7011 (30.1)			1032 (26.7)	2030 (42.4)	244 (19.3)	139 (29.0)
Discontinuers at 1-3 months Nº (%)	27 (27.3)	1948 (8.3)			346 (9.0)	806 (16.8)	97 (7.7)	54 (11.3)
Discontinuers at 1-6 months Nº (%)	35 (35.4)	3724 (16.0)			508 (13.1)	1089 (22.8)	159 (12.6)	86 (18.0)
Discontinuers at 1-12 months Nº (%)	48 (48.5)	5333 (22.9)			710 (18.4)	1402 (29.3)	211 (16.7)	117 (24.4)
**Apixaban**	**72**	**10358**			**3693**	**2728**	**2060**	**396**
Number of **discontinuers** related to Apixaban Nº (%)		1285 (12.1)			528 (14.3)	648 (23.7)	136 (6.6)	54 (13.6)
Discontinuers at 1-3 months Nº (%)	10 (13.9)	393 (3.8)			215 (5.8)	284 (10.4)	75 (3.6)	33 (8.3)
Discontinuers at 1-6 months Nº (%)	18 (25.0)	818 (7.9)			346 (9.4)	401 (14.7)	104 (5.0)	43 (10.9)
Discontinuers at 1-12 months Nº (%)	20 (27.8)	1154 (11.1)			486 (13.2)	538 (19.7)	128 (6.2)	54 (13.6)
**Rivaroxaban**	279	**11210**			**6605**	**4450**	**3606**	**1146**
Number of discontinuers related to any Rivaroxaban Nº (%)		2324 (20.7)			1256 (19.0)	1403 (31.5)	399 (11.1)	277 (24.2)
Discontinuers at 1-3 months Nº (%)	70 (25.1)	631 (5.6)			439 (6.6)	627 (14.9)	208 (5.8)	134 (11.7)
Discontinuers at 1-6 months Nº (%)	87 (31.2)	1251 (11.1)			707 (10.7)	865 (19.4)	286 (7.9)	192 (16.8)
Discontinuers at 1-12 months Nº (%)	102 (36.6)	1887 (16.8)			939 (14.2)	1094 (24.6)	369 (10.2)	246 (21.5)

Table S8. Number of New DOACs users with NVAF excluding those with other aproved DOAC indications (sensitivity analyses taking account a gap of 60 days)

	Netherlands (Mondriaan)	Denmark (NRD)	Germany (AOK NORDWEST)	Germany (Bavarian claims)	Spain (BIFAP)	Spain (SIDIAP)	United Kingdom (CPRD)	France (EGB)
**All DOACs**	**NOT APPLICABLE - NO patients with other indications**	**41708**	**NA**	**NA**	**13719**	**10287**	**6611**	**1483**
Number of **discontinuers** related to any DOACs Nº (%)		9824 (23.5)			2730 (19.9)	3573 (34.7)	737 (11.1)	339 (22.9)
Discontinuers at 1-3 months Nº (%)		2770 (6.6)			1014 (7.4)	1489 (14.5)	357 (5.4)	156 (10.5)
Discontinuers at 1-6 months Nº (%)		5362 (12.8)			1514 (11.0)	2056 (20.0)	517 (7.8)	226 (15.2)
Discontinuers at 1-12 months Nº (%)		7711 (18.5)			2070 (15.1)	2644 (25.7)	668 (10.1)	297 (20.0)
**Dabigatran**		**21885**			**3749**	**4200**	**1223**	**358**
Number of **discontinuers** related to Dabigatran Nº (%)		6602 (30.2)			1000 (26.7)	1820 (43.3)	239 (19.5)	100 (27.9)
Discontinuers at 1-3 months Nº (%)		1842 (8.4)			333 (8.9)	715 (17.0)	93 (7.6)	39 (10.9)
Discontinuers at 1-6 months Nº (%)		3509 (16.0)			493 (13.1)	971 (23.1)	155 (12.7)	61 (17.0)
Discontinuers at 1-12 months Nº (%)		5020 (22.9)			678 (18.1)	1257 (29.3)	206 (16.8)	84 (23.5)
**Apixaban**		**9669**			**3585**	**2299**	**1967**	**279**
Number of **discontinuers** related to Apixaban Nº (%)		1176 (12.2)			520 (14.5)	536 (23.3)	127 (6.5)	40 (14.3)
Discontinuers at 1-3 months Nº (%)		364 (3.8)			211 (5.9)	236 (10.3)	69 (3.5)	26 (9.3)
Discontinuers at 1-6 months Nº (%)		745 (7.7)			340 (9.5)	337 (14.7)	96 (4.9)	33 (11.8)
Discontinuers at 1-12 months Nº (%)		1048 (10.8)			479 (13.4)	443 (19.3)	119 (6.0)	40 (14.3)
**Rivaroxaban**		**10154**			**6385**	**3788**	**3421**	**846**
Number of discontinuers related to any Rivaroxaban Nº (%)		2046 (20.1)			1210 (18.9)	1217 (32.1)	371 (10.8)	199 (23.5)
Discontinuers at 1-3 months Nº (%)		564 (5.5)			470 (7.4)	538 (14.2)	195 (5.7)	91 (10.8)
Discontinuers at 1-6 months Nº (%)		1108 (10.9)			681 (10.7)	748 (19.7)	266 (7.8)	132 (15.6)
Discontinuers at 1-12 months Nº (%)		1643 (16.2)			904 (14.2)	944 (24.9)	343 (10.0)	173 (20.4)
