# Supplementary figures and images for: Adherence to Direct Oral Anticoagulants in Patients With Non-Valvular Atrial Fibrillation: A Cross-National Comparison in Six European Countries (2008–2015)
Source: Front Pharmacol. 2021 Nov 3;12:682890. doi: 10.3389/fphar.2021.682890 (PMC8596153; doi:10.3389/fphar.2021.682890)

**Figure S1. Flowcharts**

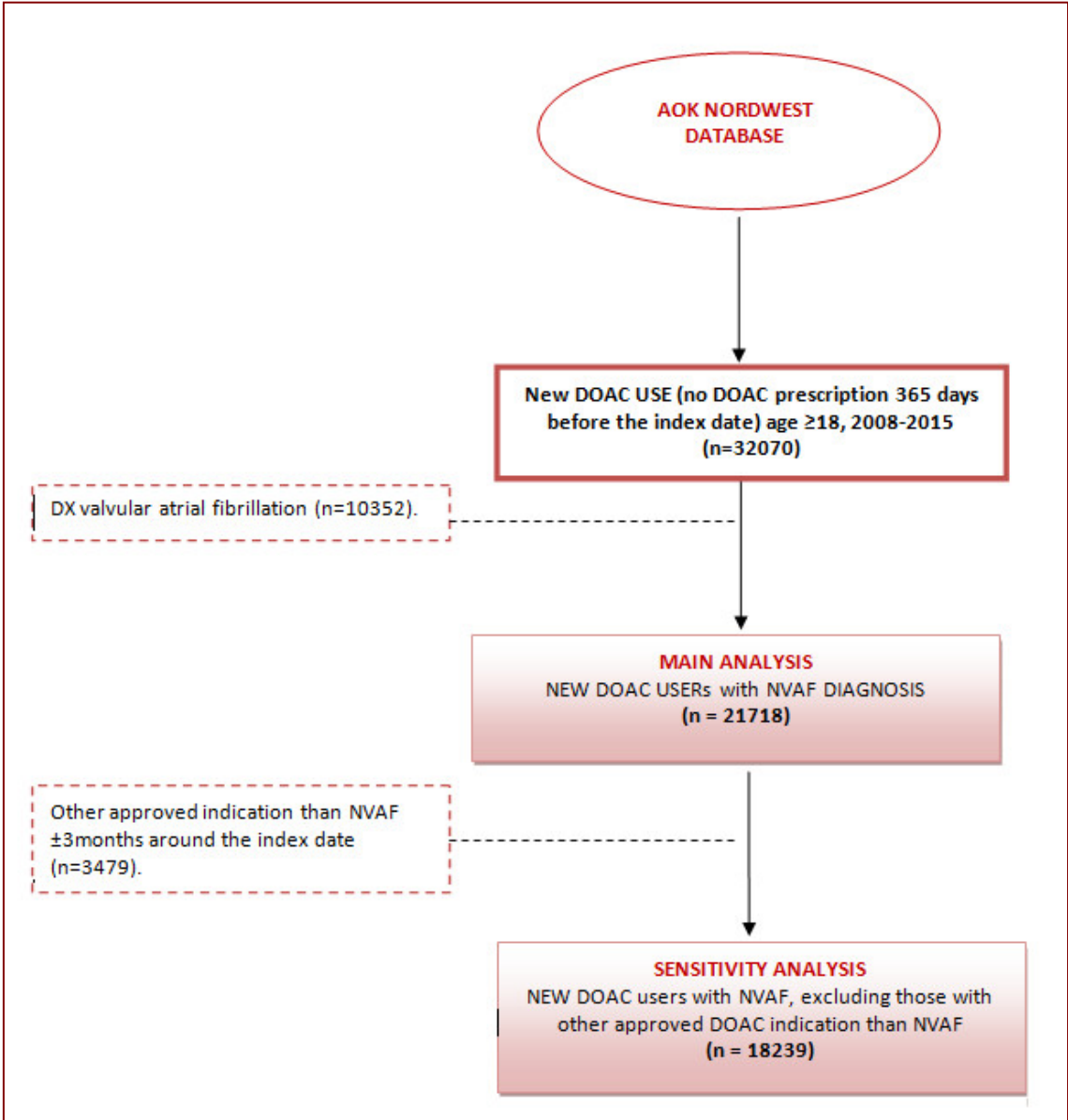

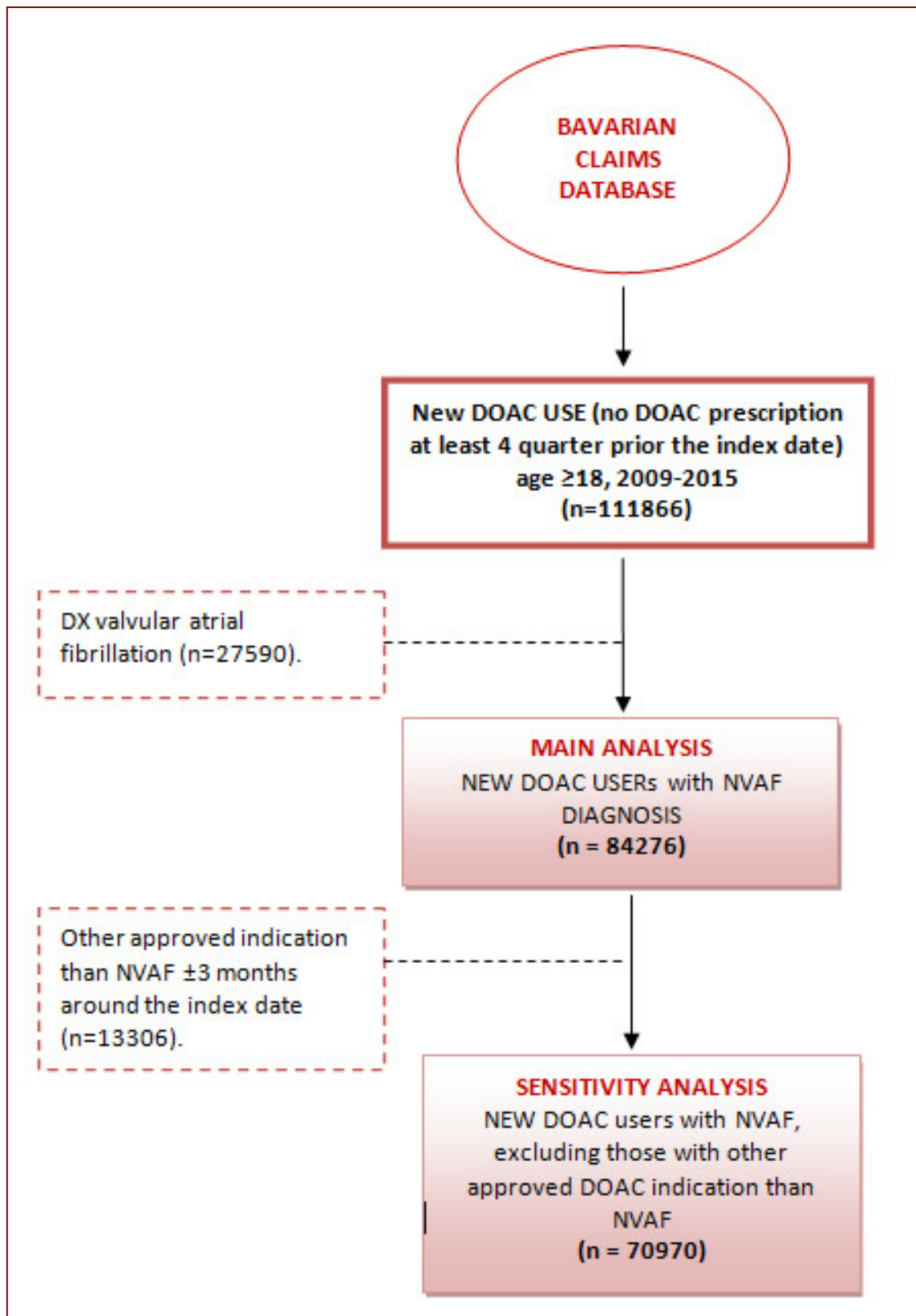

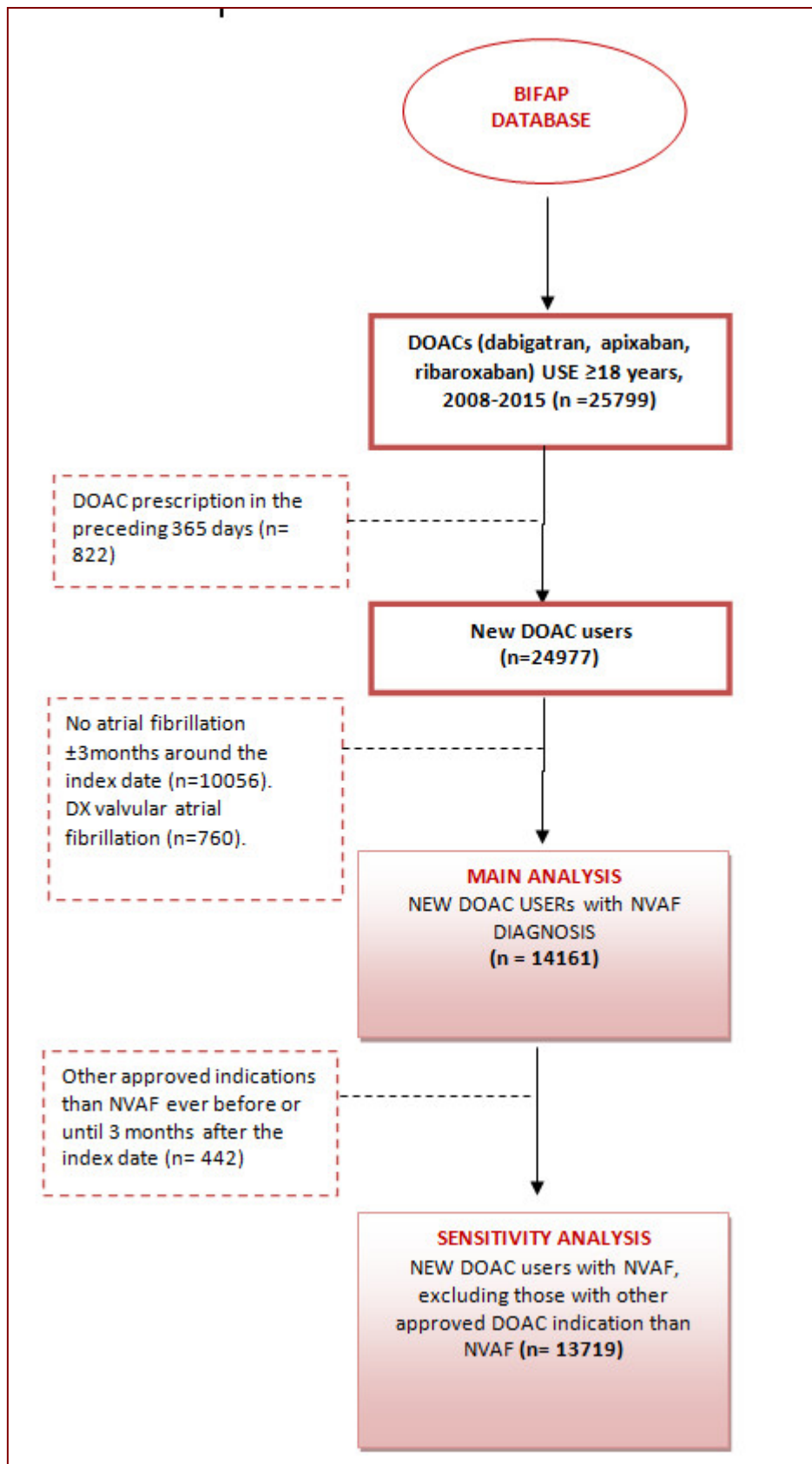

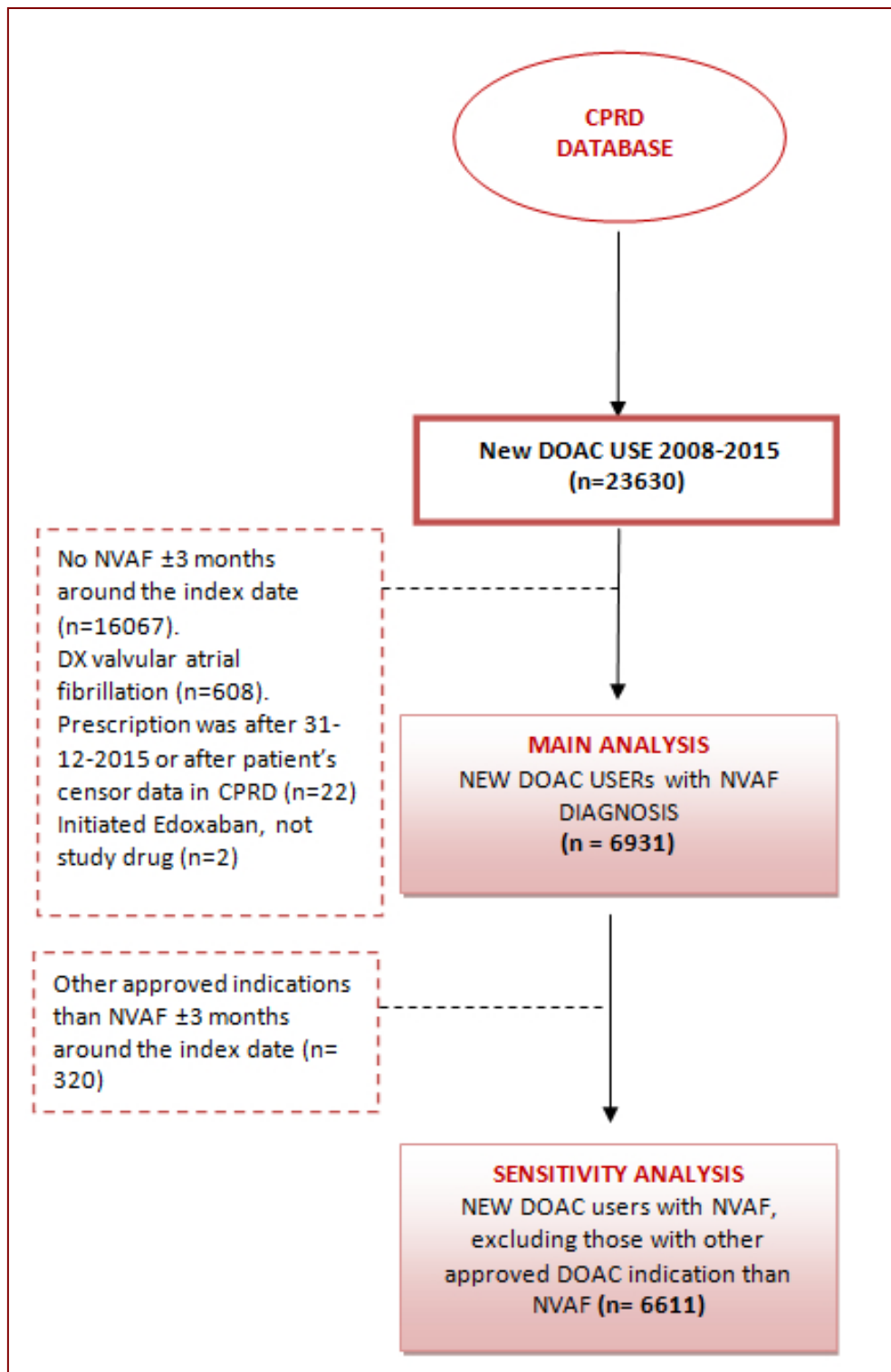

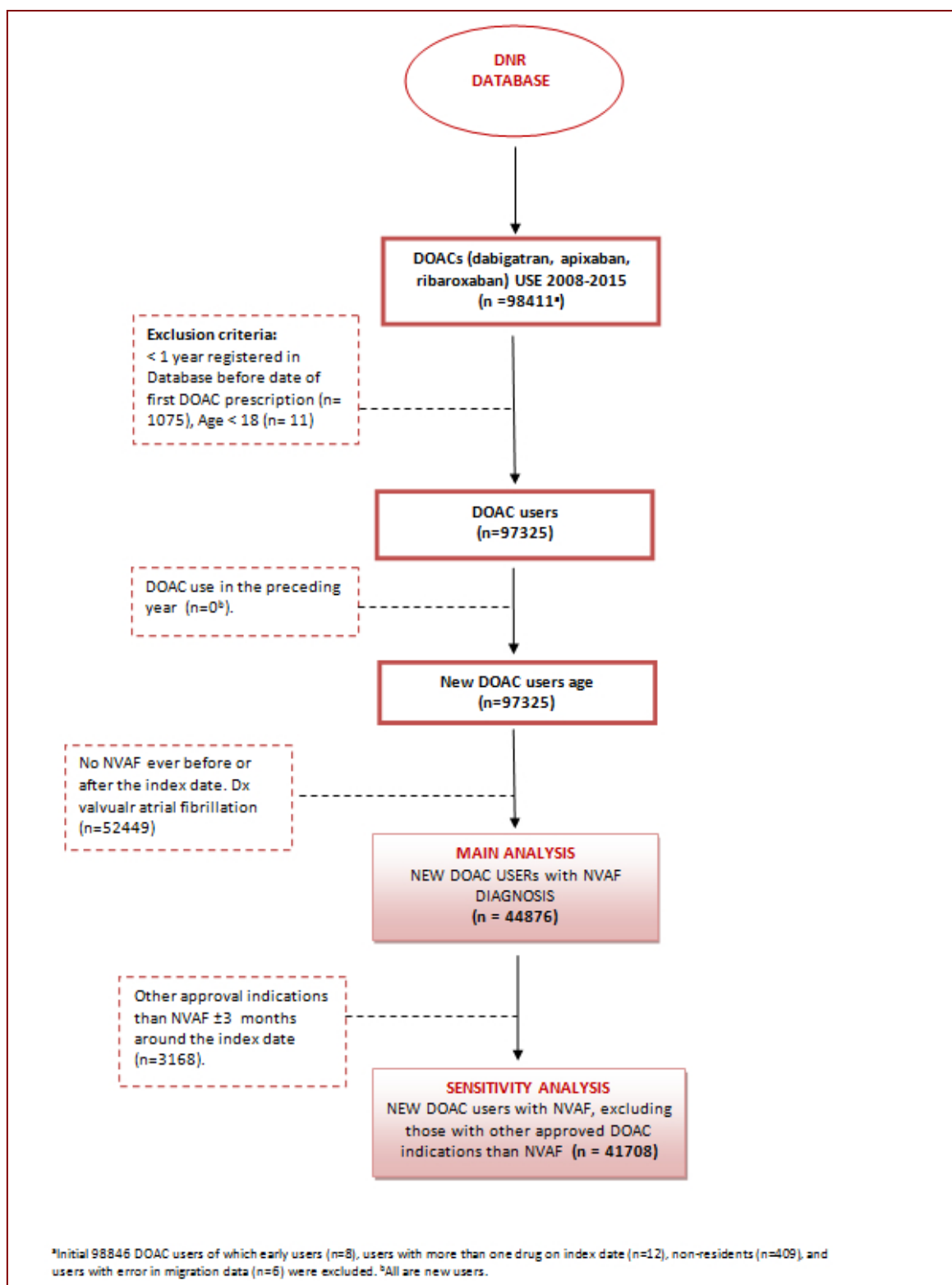



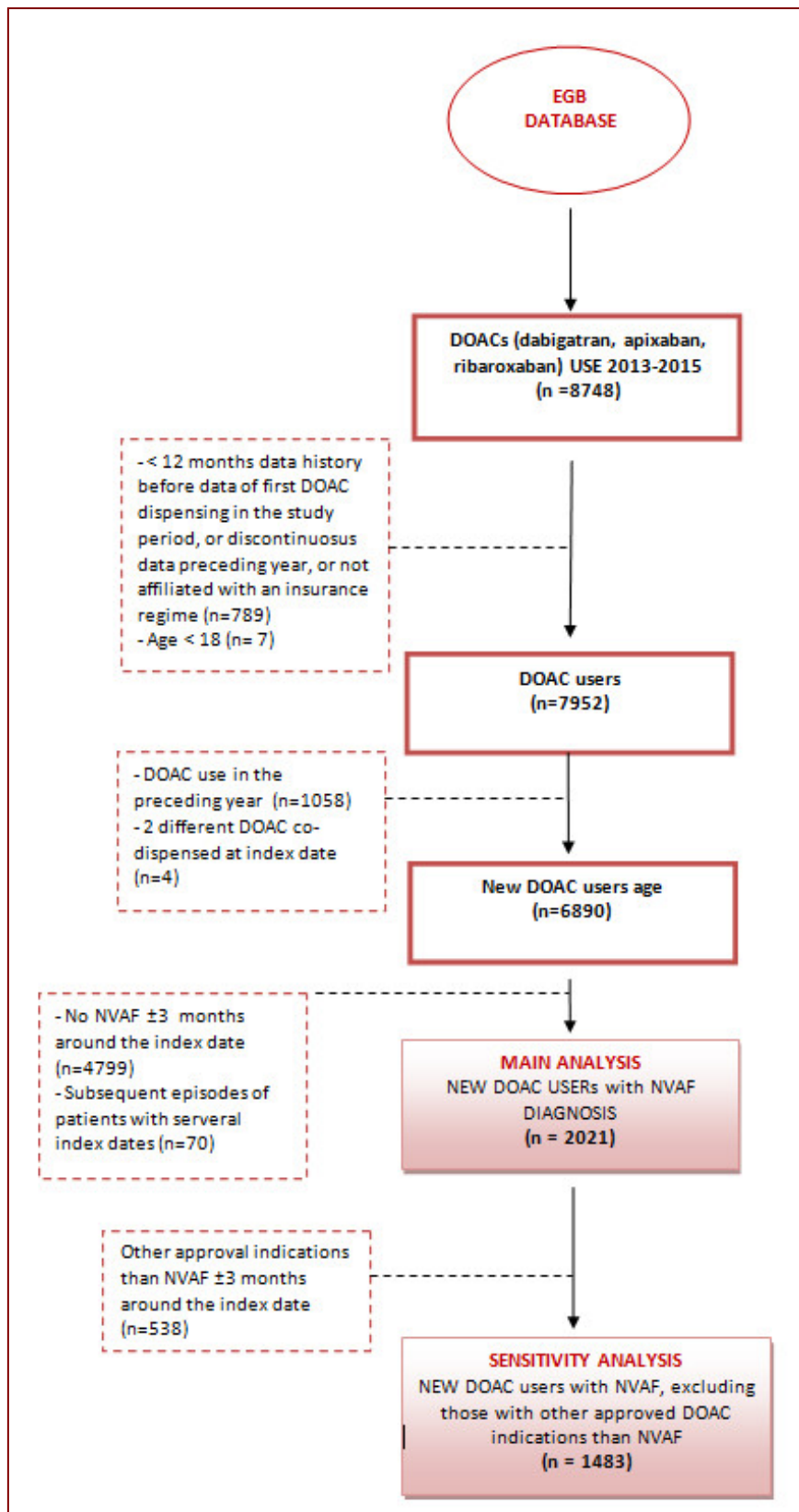

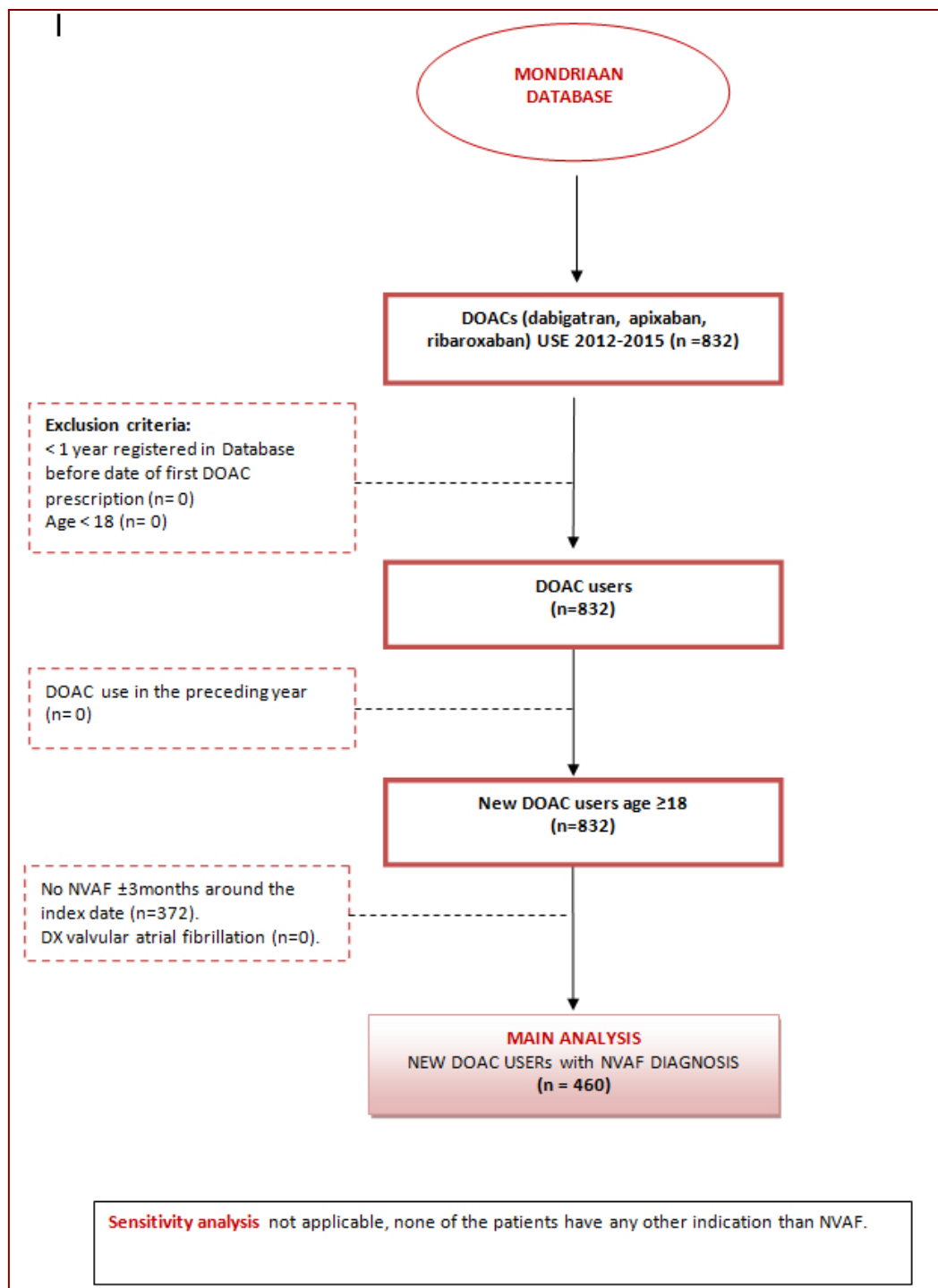

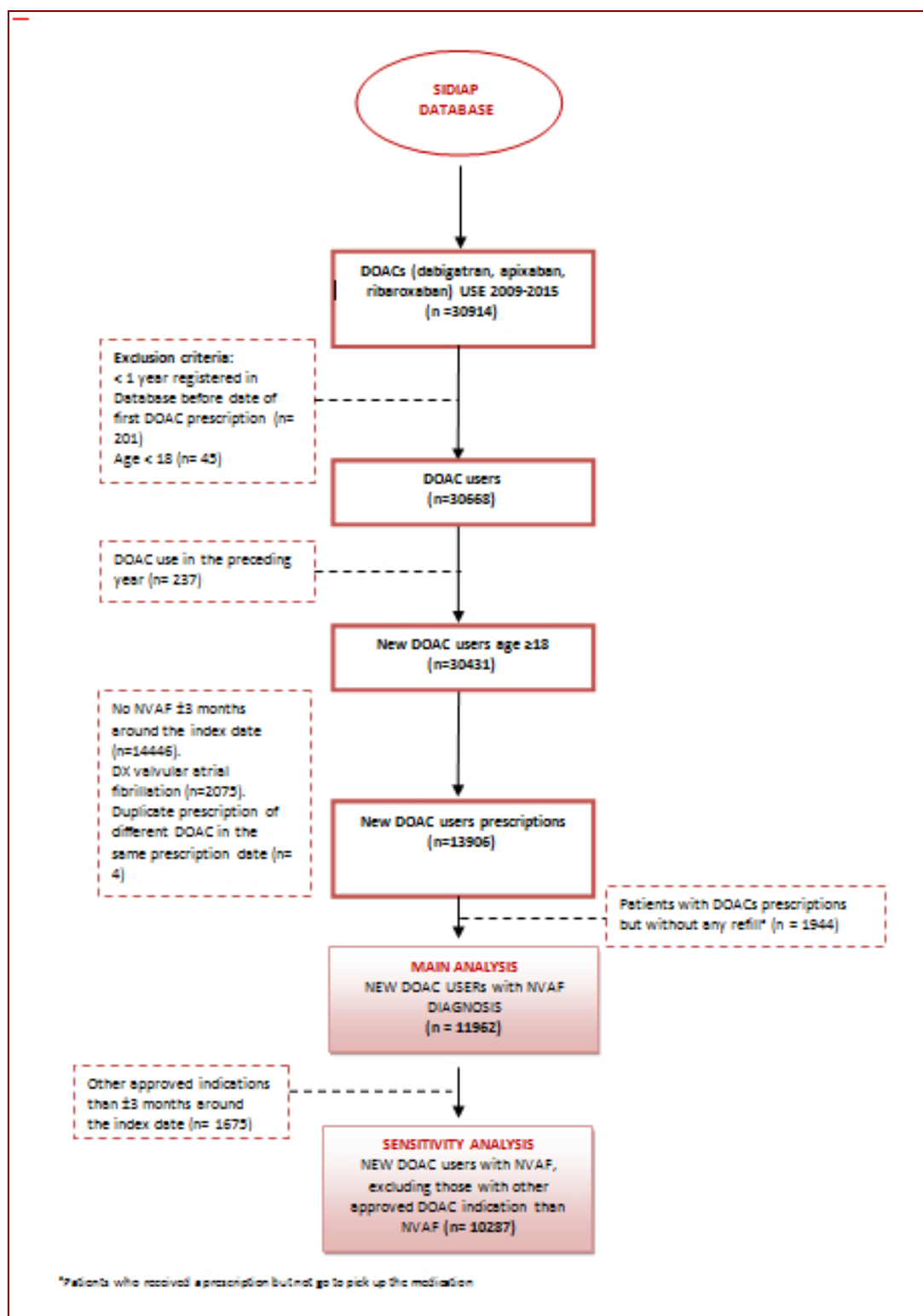

Supplement: Supplementary file 2 [file Image1.pdf]
